# Discovery of a size-record breaking green-emissive fluorophore: small, smaller, HINA†

**DOI:** 10.1039/d0sc05557c

**Published:** 2020-11-25

**Authors:** Rui Kang, Laura Talamini, Elisa D'Este, Bianca Martins Estevão, Luisa De Cola, Wim Klopper, Frank Biedermann

**Affiliations:** Institute of Nanotechnology (INT), Karlsruhe Institute of Technology (KIT) Hermann-von-Helmholtz Platz 1 76344 Eggenstein-Leopoldshafen Germany frank.biedermann@kit.edu willem.klopper@kit.edu; Institut de Science et d'Ingénierie Supramoléculaires (ISIS), Université de Strasbourg, CNRS 8Rue Gaspard Monge 67083 Strasbourg France decola@unistra.fr; Optical Microscopy Facility, Max Plank Institute for Medical Research Jahnstraße 29 D-69120 Heidelberg Germany; Institute of Physical Chemistry (IPC), Karlsruhe Institute of Technology (KIT) Fritz-Haber-Weg 6 76131 Karlsruhe Germany

## Abstract

Astonishingly, 3-hydroxyisonicotinealdehyde (HINA) is despite its small size a green-emitting push–pull fluorophore in water (QY of 15%) and shows ratiometric emission response to biological relevant pH differences (p*K*_a2_ ∼ 7.1). Moreover, HINA is the first small-molecule fluorophore reported that possesses three distinctly emissive protonation states. This fluorophore can be used in combination with metal complexes for fluorescent-based cysteine detection in aqueous media, and is readily taken up by cells. The theoretical description of HINA's photophysics remains challenging, even when computing Franck–Condon profiles *via* coupled-cluster calculations, making HINA an interesting model for future method development.

## Abstract 2

Astonishingly, 3-hydroxyisonicotinealdehyde (HINA) is despite its small size a green-emitting push–pull fluorophore in water (QY of 15%) and shows ratiometric emission response to biological relevant pH differences (p*K*_a2_ ∼ 7.1).

## Graphical abstract

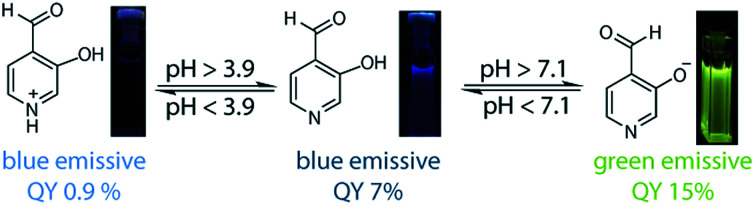

## Introduction

**Scheme 1:**
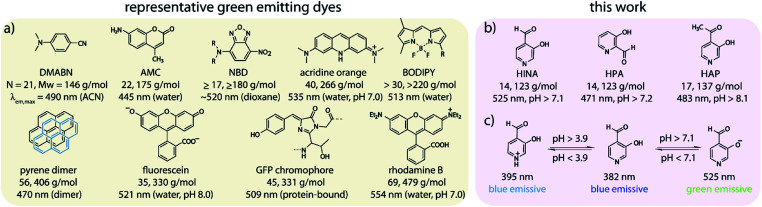
Chemical structures, abbreviations, atom numbers (*N*), molecular weights (*M*_w_) and the peak maximum of emission (*λ*_em, max_) of (a) representative green-emitting dyes, and of (b) herein investigated formyl/acetyl-hydroxy-pyridines. (c) (De)protonation equilibria of HINA in water.

Fluorescence is both fundamentally fascinating and practically relevant. Countless examples of fluorescent compounds have been described and hundreds of dyes have found commercial use.^1–4^ Decade-long debates were spurred about the origin of the dual fluorescence of DMABN, a small, weakly emissive push–pull system (Scheme 1a).^5–7^ For comparison, the GFP chromophore is, discounting the surrounding protein pocket, one of the smallest green-emitting fluorophores of biological origin.^8^ However, synthetic protein-free GFP analogues suffer from emission quenching by protic solvents.^8–10^ Structurally related polymethine or squarylium push–pull dyes consist of at least two methylene-bridged aromatic moieties.^11–15^ Nitrobenzoxadiazole-derivatives (NBDs) are typical push–pull fluorophores that display good quantum yields (QY) of ∼10% in hydrophobic environments but are not emissive in water.^16–18^ Thus, rhodamines, fluoresceins, coumarins, BODIPYs (Scheme 1a) and other polyaromatic dyes are commonly utilized as water-soluble green-emitting dyes.^11–13,19,20^ However, motivated by the need for smaller, non-disturbing fluorescent labels,^16,21^ the search for compact fluorophores that are applicable to aqueous media continues.^13,22^ Furthermore, luminescent compounds that possess a rich photophysical behaviour also provide valuable test cases for theoretical electron-dynamics method development.^23–25^

We have stumbled over the unexpected emission of 3-hydroxyisonicotinealdehyde (HINA, see Scheme 1b) in aqueous media. Surprisingly, while the absorbance properties of HINA have been known for decades,^26–28^ its unique fluorescent properties were completely overlooked.^29^ HINA consists of only 14 atoms (123 g mol^−1^); for comparison, the similar-sized fluorophores naphthalene and azulene (both 18 atoms, 128 g mol^−1^) are blue emitting, *λ*_em,max_ = 327 and 373 nm in methanol, respectively.^30–32^ Typical green-emitting dyes are much larger in terms of atom number, molecular weight and size than HINA, see Scheme 1a.

## Results

**Fig. 1:**
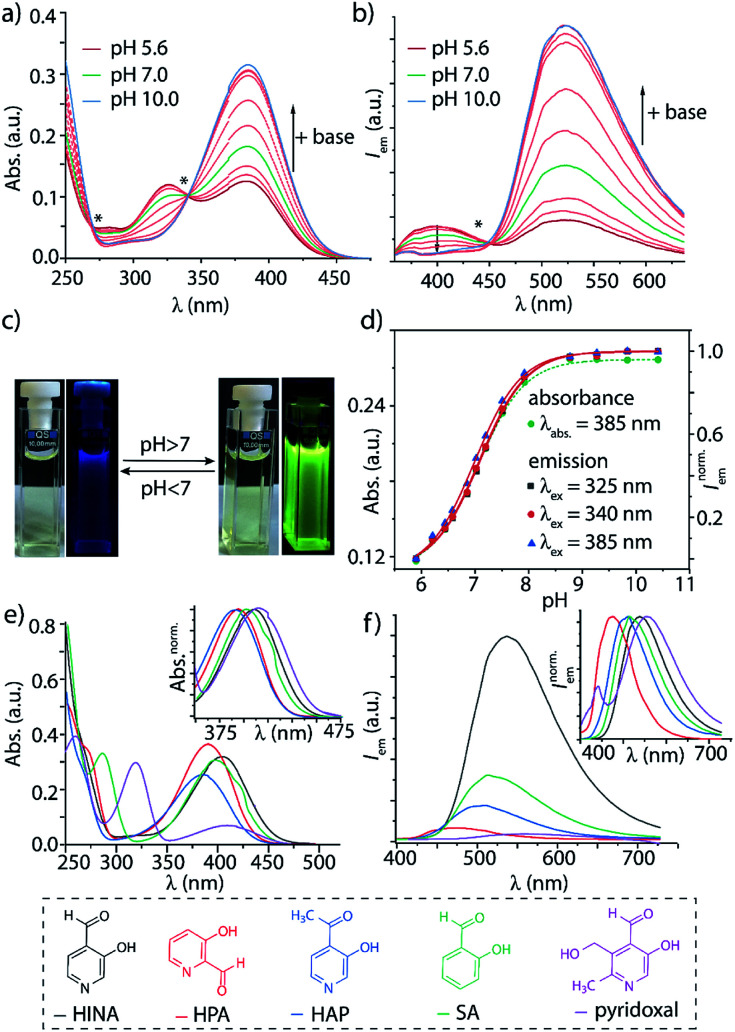
Titration of an aqueous solution of HINA (50 μM) with NaOH_aq_ monitored by (a) absorbance and (b) emission spectroscopy (*λ*_ex_ = 330 nm). p*K*_a2_ = 7.05 ± 0.01 and p*K*_a2_ = 6.98 ± 0.01 for absorbance- and emission (*λ*_ex_ = 385 nm)-based titration. * Isosbestic or isoemissive point. (c) Photographic images of HINA for its neutral (left) and anionic form (right) under ambient light and UV irradiation. (d) Absorbance (*λ*_abs_ = 385 nm) and [0,1]-normalized emission intensity (*λ*_ex_ = 325, 340 and 385 nm, *λ*_em_ = 533 nm) as a function of pH. Fits shown as dashed green (absorbance) and solid red (emission) lines. Absorbance (e) and emission (f) spectra in water at pH ≥ 9.5 of HINA, HPA, HAP, SA and pyridoxal. Insets show the normalized absorbance and emission spectra at their maximum absorbance and emission wavelength, respectively.

Systematic investigations by absorbance and emission spectroscopy were carried out to elucidate the photophysical behaviour of HINA (Fig. 1). At 3.8 < pH < 7.0, HINA occurs in its neutral form and is blue emissive (*λ*_em,max_ = 382 nm) with a respectable QY of 7% (Table 1). Upon addition of NaOH, a new absorbance centered at 385 nm arose while the band at 325 nm decreased (Fig. 1a). Simultaneously, the blue emission of neutral HINA vanished and an even stronger green emission (*λ*_em,max_ = 525 nm) with a QY of 15% appeared;^33^ the corresponding colour change can be seen by the naked eye (Fig. 1b and c). The p*K*_a_ value for deprotonation of HINA was obtained by absorbance-based titration, p*K*_a2_ = 7.05 ± 0.01, and agreed with the value from pH-meter recordings, see Fig. 1d and S10.† Coincidentally, this p*K*_a_ value lies perfectly in the biologically relevant range. Interestingly, very similar p*K*_a2_ values (∼7) were obtained by fluorescence-based titrations regardless of the excitation wavelength, *i.e.* by exciting the neutral form of HINA, the anion or both at an isosbestic point. This indicates that deprotonation of the neutral form of HINA is not a significant process during the lifetime of its excited state^34^ despite its largely negative 
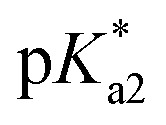
 value estimate of −5.5, which was obtained by a Förster cycle analysis (see the ESI†).^35,36^ Unlike many other hydroxarenes such as phenols and naphthols,^34,37^ HINA does not function as a photoacid. HINA becomes *N*-protonated below pH 3.9, where it remains fluorescent, albeit with a low QY of 0.9%. The emission band of cationic HINA is centred at 395 nm, and thus surprisingly bathochromically shifted compared to neutral HINA (Fig. S1† and Table 1). Conversely, the absorbance spectra showed the expected hypsochromic shift upon addition of acid. The emission lifetimes of the cationic, neutral and anionic forms of HINA identified them as fluorophores (Table 1). Indeed, HINA is a fascinating emissive dye that occurs in three distinct emissive forms. We are unaware of any other small fluorophore with this property.

**Table 1:** Photophysical properties and p*K*_a_ values of HINA, HPA and HAP in aerated water at 25 °C

	Cationic form at pH < p*K*_a1_			Neutral form at p*K*_a1_ < pH < p*K*_a2_			Anionic form at pH > p*K*_a2_			p*K*_a1_, p*K*_a2_ in H_2_O^h^
	*λ*_abs_ (nm) & [log *ε*]^a^	*λ*_em_ b (nm) & QY^c^	*τ* d (ns)	*λ*_abs_ (nm) & [log *ε*]^a^	*λ*_em_ e (nm) & [QY]^f^	*τ* d (ns)	*λ*_abs_ (nm) & [log *ε*]^a^	*λ*_em_ g (nm) & [QY]^f^	*τ* d (ns)	
HINA	286 [3.71]	395 [0.9%]	0.2	325 [3.32]	382 [7.0%]	0.9	385 [3.74]	525 [15.0%]	1.0	3.9, 7.1
HPA	285 [3.84]	395 [0.4%]	0.2	316 [3.63]	376 [1.2%]	0.4	369 [3.87]	484 [1.4%]	0.2	3.2, 7.2
HAP	318 [3.64]	n.a.^i^	—	329 [3.53]	410 [0.1%]	0.2	365 [3.72]	484 [0.8%]	0.9	2.6, 8.1

a Absorbance peak maximum and logarithmic extinction coefficient.

b Emission peak maximum, *λ*_ex_ = 285 nm.

c Emission quantum yield, *λ*_ex_ = 300 nm.

d Emission lifetime, *λ*_ex_ = 373 nm.

e Emission peak maximum, *λ*_ex_ = 330 nm.

f Emission quantum yield, *λ*_ex_ = 370 nm.

g Emission peak maximum, *λ*_ex_ = 380 nm.

h From pH titration experiments. Estimated errors ± 0.1 from different methods, see the ESI.

i Very weak emission.

Strongly solvent-dependent absorbance and emission spectra of HINA were observed. For instance, the characteristic ∼340 nm absorbance maxima of neutral HINA and a weak blue emission was found in anhydrous acetonitrile (ACN). In contrast, a long-wavelength absorbance band and green emission appeared when water was added to ACN (Fig. S13†). Indeed, the emission of HINA is centred at ∼400 nm in all anhydrous organic solvents tested (DMSO, MeOH, ethylene glycol, Fig. S14–S18†), but shifts to the 450–600 nm range upon addition of water. Generally, the emission quantum yield of HINA increases upon deprotonation and is higher in polar protic than in polar aprotic solvents, *e.g.* reaching up to 24% in basified methanol (Table S8†).

In aqueous environments, the aldehyde moiety of HINA equilibrates with its hydrate structure, as confirmed by UV/Vis^38^ and NMR experiments (ESI, Fig. S42–S46†). At first, we wondered if one of the hydrated forms of HINA is the green-emitting species. However, fluorescence in the 450 to 700 nm wavelength region was also detected when adding anhydrous base or anion-stabilising urea to a solution of HINA in organic solvents (see Fig. S16–S18†), confuting hydrate-contributions. Unlike pyrenes (Scheme 1a) or other aggregation-induced emitting (AIE) systems,^3,24,39^ HINA emission is not caused by aggregate formation (Fig. S27 and S28†).

The photophysical properties of 3-hydroxypicolinealdehyde (HPA, an isomer of HINA), of 3-hydroxy-4-acetylpyridine (HAP, a ketone analogue of HINA), of salicylaldehyde (SA, a phenyl analogue of HINA) and of pyridoxal (a biological hydroxy-formyl-pyridine) were evaluated to obtain insights into the photophysical mechanism, see Fig. 1e, f and Table 1. HPA showed spectroscopic features that were similar to that of HINA, but its absorbance and emission bands for both the neutral and anionic forms are hypsochromically shifted. Moreover, HPA is a much weaker emitter than HINA. The ketone HAP is also poor emissive in its neutral form and as an anion. HAP requires a higher pH for deprotonation than HINA or HPA. Notably, HAP does not form hydrates in water as was confirmed by ^1^H NMR experiments (Fig. S55†), providing additional evidence that these are not the blue and green-emitting species. Like for HINA, absorbance and emission-based titrations of HPA and HAP yielded essentially overlaying pH–property plots (Fig. S6–S9†). Again, effective deprotonation of the neutral form of the hydroxy-pyridines apparently does not occur during the lifetime of the excited state despite the strong acidity of the excited state 
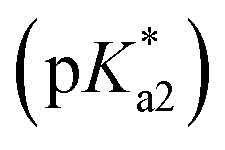
 of the fluorophores that was estimated by Förster's method (see ESI†).

7-Azaindole (7AI, 15 atoms) and SA (15 atoms) are to the best of our knowledge the smallest green-emitting fluorophores that have been reported so far, but they require the use of an unphysiological basic pH of >13.5 and >8.4, respectively.^40–42^ SA is structurally closely related to HINA (14 atoms) but shows despite its more electron-rich phenyl ring somewhat counterintuitively a hypochromic shifted absorbance and emission spectra (Fig. 1e and f). Compared to HINA, SA is an inferior fluorophore because its neutral form is not fluorescent and its anionic form is seven times weaker emissive (QY = 2% at pH 10.0). Pyridoxal is both significantly larger in size and much less emissive than the other formyl-hydroxy-pyridines.

The character of the emitting electronic states has been investigated by computing the Franck–Condon profiles through coupled-cluster (CC) calculations with the def2-TZVPPD basis set, see Table S20.† For the anionic form of HINA, the predicted absorption and emissions bands, and the Stokes shift are in good agreement with the experimental findings (Fig. S72 and Tables S20–S22†). The n → π* transition displayed an almost zero oscillator strength, whereas that of the π → π* transition is four orders of magnitude larger, resulting in computed RGB values (Table S22†) that agreed well with the visually observed green emission. Also, the relative emission wavelength maxima trends between HINA, HPA and SA were correctly predicted (Table S20†). Nevertheless, the theoretical description of HINA and its analogues is not simple, despite the small size of these fluorophores. In fact, electronic states which are very close in energy were present and it was found that vibronic effects^23,24,43^ are crucial for the description of the photophysical properties.^44^

Very large discrepancies with the experiments were encountered when computing the emission transitions for the neutral forms of HINA and its analogues. Indeed, an excited-state intramolecular proton-transfer process (ESIPT)^23,45–47^ likely occurs for these substances (Fig. S73†). An intramolecular ESIPT may also explain the large discrepancies seen between the expected pH-dependent deprotonation of the excited state based on calculated 
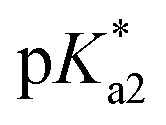
 values, and the “ground-state like” protonation status observed in fluorescence experiments. Thus, explicit theoretical treatment of the electron dynamics is required for computing the photophysical properties of (neutral) HINA, which is beyond the scope of this work. HINA and its analogues represent challenging test cases for advanced theoretical studies and method developments of the nuclear and electronic dynamics, because they combine fascinating photophysical properties with advantages for carrying out the computations (small number of atoms, electrons, and conformers).

The large observed Stokes shifts, *e.g.* 6700, 4600 and 6900 cm^−1^ for protonated, neutral and deprotonated HINA characterize it as a push–pull dye, *e.g.* ∼5000 cm^−1^ for NBDs and ∼8400 cm^−1^ for recently reported benzoxazole-thiophenes^48^ and stilbenes.^17^ This raises the question if more compact but emissive push–pull fluorophores are even conceivable because single aryl ring-based HINA, HPA and SA already feature some of the smallest but most powerful donor and acceptor moieties, *i.e.* –O^−^ with a donor resonance const. R^+^ of −2.04, and –CHO with an acceptor resonance const. R^−^ of 0.70.^49^ Note that similarly-sized cyanophenols (R^−^ = 0.49 for –CN) are not emissive.

Unlike most other push–pull fluorophores, HINA shows a good emission quantum yield in water, which makes it attractive for applications. For instance, it may be used as ratiometric, fluorescent pH indicator dye because its protonated, neutral and deprotonated forms display characteristic emission profiles. Complementary to many other green emitting dyes that interact with hydrophobic species, HINA's high hydrophilicity diminishes its binding to β-cyclodextrin and cucurbit[7]uril as macrocyclic hosts, to serum albumin as a carrier protein, and to polymethacrylate- and polystyrene surfaces.

**Fig. 2:**
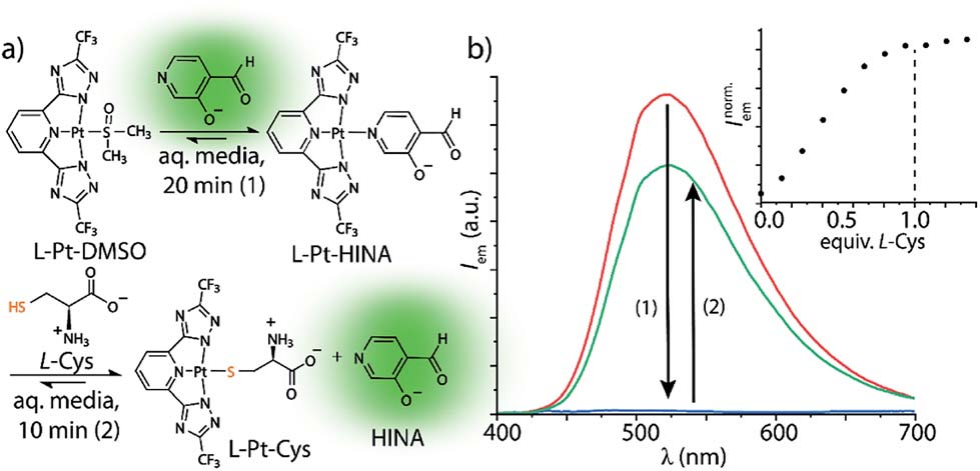
(a) HINA can be reacted with a Pt(ii)-precursor to form a non-emissive l-Pt-HINA complex with a binding constant of *K*_a_ ∼ 10^6^ M^−1^, see ESI.† Upon addition of l-Cys, the HINA ligand is displaced and becomes emissive. (b) Emission spectra of HINA (50 μM, red line) upon addition of l-Pt-DMSO (50 μM, blue line), and subsequent addition of l-Cys (50 μM, green line). The inset shows a titration of l-Pt-HINA (50 μM) with l-Cys, 0 to 73 μM (*λ*_em_ = 533 nm). All the spectra were obtained in 25 mM NaHCO_3_ buffer (pH 9.5) containing 0.9 mM CTAB.

HINA can also function as a fluorescent indicator in supramolecular sensing assays: we observed that HINA coordinates to Pt- and Pd-complexes, resulting in a full fluorescence switch off (see Fig. 2). From those complexes, the HINA ligand can be readily displaced by a stronger ligand, *e.g.* thiols, yielding in an emission switch-on response in aqueous and organic media. In fact, micromolar concentrations of l-cysteine (l-Cys) can be rapidly (∼10 min) detected through a fluorescence-based indicator-displacement assay in that way (Fig. 2b). Analogously, HINA can be displaced from a Pd-complex by pyridine (Fig. S65†). We believe that HINA-capped metal–organic building blocks could be reacted on demand with stronger ligands to form metal–organic cages or -frameworks,^50–52^ thereby providing an *in situ* fluorescence response for monitoring reactions in real time. Noteworthy, HINA also forms covalent conjugates with l-Cys in aqueous media (Fig. S66–S68†), *via* the formation of a 1,3-thiazolidine ring, which is reminiscent to other aldehyde moiety-containing cysteine-reactive probes.^53–55^ However, like for most reported CHO-based probes, a large excess of l-Cys and long reaction times are needed, making it less attractive for the sensing applications than the HINA-based indicator displacement assay introduced above.

**Fig. 3:**
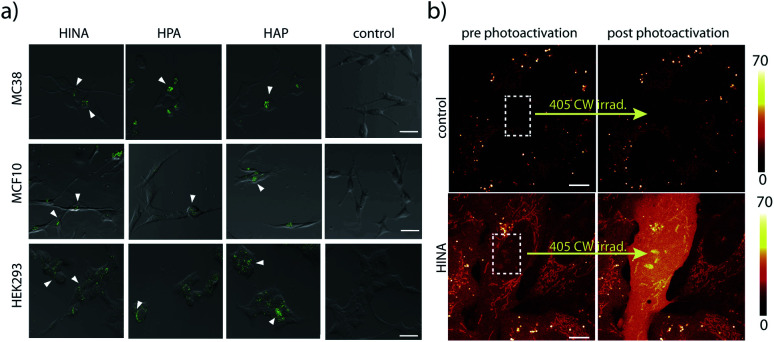
(a) Microscopy images of the internalization of HINA, HPA, and HAP in MC38, (top row) MCF10 (middle row) and HEK293 (bottom row) cell line after 1 hour of incubation at the concentration of 100 μM. The green signal (white arrowhead) is related to dye molecules and it is mainly localized in the perinuclear region of cells. The control experiment (untreated with dyes) did not show any specific signal (left panel). Scale bar: 20 μm. (b) Confocal images before and after photoactivation of U2OS cells incubated with 2 mM HINA followed by washing (bottom row), and for the control without dye. Dashed boxes indicate the area that was irradiated with a CW 405 light source for three seconds. Colour bars and corresponding minimum and maximum number of counts in displayed in the image are on the right. Scale bars: 10 μm.

HINA, HPA and HAP readily permeate through biological membranes^19,56^ as was confirmed for different cancerous and non-cancerous cell lines, see the ESI.† Preliminary results indicate that they localize in the perinuclear region of the cells (Fig. 3a) and the cell toxicity level of the dyes is low (Fig. S69†). Interestingly, a rapid fluorescence photoactivation of HINA-treated cells was observed within seconds during the fluorescence imaging experiments (Fig. 3b and Videos in the ESI†). A similar behaviour was seen in control experiments upon irradiation of HINA solutions with a strong light source, and in the presence of hydrogen peroxide (Fig. S71†).

## Conclusions

In conclusion, 3-hydroxyisonicotinealdehyde (HINA) has been identified as the smallest known green fluorescent dye, and may be reaching the fundamental size-limit. Uniquely, HINA occurs in three different protonation states that are each distinctly fluorescent. The QY of HINA in water is surprisingly high, despite being a push–pull chromophore. HINA's commercial availability, and its favourable photophysical properties (large Stokes shift, pH-dependent ratiometric emission properties that are switching in biorelevant pH 7 regime) will enable future applications, ranging from its use as a fluorescent dye to its function as an indicator in supramolecular assays. The synthesis and investigation of additional hydroxyl-functional pyridine-aldehydes and -ketones will lead to the discovery of novel green-emissive labels with improved photophysical properties. These dyes will like HINA provide excellent test cases to evaluate theoretical predictions of emission spectra using highly advanced computational methods capable of considering both vibronic effects and excited-state intramolecular proton-transfer process (ESIPT).

## Conflicts of interest

There are no conflicts to declare.

## Supplementary Material

SC-012-D0SC05557C-s001
