# Supplementary material for: Discovery of a size-record breaking green-emissive fluorophore: small, smaller, HINA
Source: Chem Sci. 2020 Nov 25;12(4):1392–7. doi: 10.1039/d0sc05557c (PMC8179180; doi:10.1039/d0sc05557c)
Supplement: SC-012-D0SC05557C-s001 [file SC-012-D0SC05557C-s001.pdf]

Electronic Supplementary Material (ESI) for Chemical Science.
This journal is © The Royal Society of Chemistry 2020

Electronic Supplementary Information

Discovery of a size-record breaking green-emissive fluorophore: Small, smaller, HINA

Rui Kang,[a] Laura Talamini,[b] Elisa D'Este,[c] Bianca Martins Estevão,[b] Luisa De Cola,*[a, b] Wim Klopper,*[d] and Frank Biedermann*[a]

Table of Contents

1. General Information ... 3
1.1. Abbreviations ... 3
1.2. Experimental details ... 4
2. pH titration experiment ... 4
2.1. pH titration photophysical experiment ... 4
2.1.1. Absorbance and emission spectra ... 5
2.1.1.1. 3-hydroxy-isonicotinic aldehyde (HINA) ... 5
2.1.1.2. 3-hydroxy-picolin aldehyde (HPA) ... 6
2.1.1.3. 3-hydroxy-4-acetyl pyridine (HAP) ... 7
2.1.2. pK_a fitting ... 7
2.1.2.1. 3-hydroxy-isonicotinic aldehyde (HINA) ... 7
2.1.2.2. 3-hydroxy-picolin aldehyde (HPA) ... 8
2.1.2.3. 3-hydroxy-4-acetyl pyridine (HAP) ... 10
2.2. pK_a value determination by pH meter ... 11
2.2.1. 3-hydroxy-isonicotinic aldehyde (HINA) ... 11
2.2.2. 3-hydroxy-picolin aldehyde (HPA) ... 12
2.2.3. 3-hydroxy-4-acetyl pyridine (HAP) ... 12
2.3. Calculation of excited-state acidities (pK_{a2}*) of HINA, HPA and HAP using Förster cycle analysis ... 13
3. Absorbance and emission spectra in organic solvents and solvent mixtures ... 14
3.1. 3-hydroxy-isonicotinic aldehyde (HINA) ... 14
3.1.1. Spectra of HINA in acetonitrile (ACN) upon addition of water. ... 14
3.1.2. Spectra of HINA in dimethyl sulfoxide (DMSO) upon addition of water ... 15
3.1.3. Spectra of HINA in DMSO upon addition of anhydrous base or urea ... 16
3.1.4. Spectra of HINA in MeOH upon addition of anhydrous base ... 16
3.1.5. Spectra of HINA in ethylene glycol (EG) upon addition of anhydrous base ... 17
3.1.6. Spectra of HINA in hexafluoro-2-propanol (HFP) ... 17
3.2. 3-hydroxy-picolin aldehyde (HPA) ... 18
3.2.1. Spectra of HPA in ACN upon addition of water ... 18
3.2.2. Spectra of HPA in MeOH upon addition of anhydrous base ... 18
3.2.3. Spectra of HPA in EG upon addition of anhydrous base ... 19
3.3. 3-hydroxy-4-acetyl pyridine (HAP) ... 19
3.3.1. Spectra of HAP in ACN upon addition of water ... 19
3.3.2. Spectra of HAP in DMSO upon addition of anhydrous base ... 20
3.3.3. Spectra of HAP in MeOH upon addition of anhydrous base ... 20
3.3.4. Spectra of HAP in EG upon addition of anhydrous base ... 21
4. Emission quantum yields in organic solvents ... 21
5. Concentration-depencency investigations ... 22
5.1. Concentration increase of HINA in buffer ... 22

5.2. Concentration decrease of HINA in buffer 22
6. Salicyladehyde (SA) measurement 23
7. Pyridoxal measurement 23
8. Purity determination of HINA by analytical HPLC measurements 24
9. NMR measurement 25
9.1. 3-hydroxy-isonicotinic aldehyde (HINA) 25
9.1.1. DMSO-d_6 25
9.1.2. NMR in a solvent mixture of DMSO-d_6 with D_2O 28
9.1.3. Acetonitrile-d_3 (ACN-d_3) 29
9.1.4. Deuterium oxide (D_2O) 30
9.1.5. Deuterium oxide (D_2O) under basic conditions 33
9.1.6. hexafluoro-2-propano (HFP) 34
9.2. 3-hydroxy-picolin aldehyde (HPA) 35
9.2.1. ACN-d_3 35
9.2.2. Deuterium oxide (D_2O) 36
9.3. 3-hydroxy-4-acetyl pyridine (HAP) 37
9.3.1. ACN-d_6 37
9.3.2. Deuterium oxide (D_2O) 39
10. HINA indicator displacement assay with Platinum (Pt) complex 40
10.1. Assay in organic solvent 40
10.2. Assay in aqueous media 41
10.2.1. MS spectra 41
10.2.2. Binding constant determination 43
10.3. NMR spectra 44
11. HINA indicator displacement assay with palladium (Pd) complex 47
12. Cysteine sensing with HINA in aquous media 48
12.1. spectra measurement 48
12.2. NMR spectra 49
13. Cell experiment 50
14. Photoactivation experiments 51
14.1. Photoactivation in cell 51
14.2. Photoactivation test in solvent 52
15. Computational results 53
16. References 57

1. General Information

1.1. Abbreviations

HINA	3-hydroxy-isonicotinic aldehyde
HPA	3-hydroxy-picolin aldehyde
HAP	3-hydroxy-4-acetyl pyridine
SA	salicylaldehyde
ACN	acetonitrile
DMSO	dimethyl sulfoxide
MeOH	methanol
EG	ethylene glycol
HFP	1,1,1,3,3,3-hexafluoro-2-propanol
D_2O	deuterium oxide
ESIPT	excited-state intramolecular proton-transfer
L-Cys	*L*-cysteine

1.2. Experimental details

All solvents were used as received from Aldrich or FluoroChem without any further purification. All chemicals were purchased and used as received. ^{1}H NMR spectra were recorded on a Bruker Avance 500 spectrometer. ^{1}H NMR chemical shifts (δ) are given in ppm and refer to residual protons on the corresponding deuterated solvent. All deuterated solvents were used as received without any further purification.

Absorption spectra were measured on a Jasco V-730 double-beam UV-vis spectrophotometer and baseline corrected.

Steady-state emission spectra and time resolved emission profiles were recorded on a Jasco FP-8300 fluorescence spectrometer equipped with a 450 W xenon arc lamp, double-grating excitation, emission monochromators. Emission spectra were corrected for source intensity (lamp and grating) and the emission spectral response (detector and grating) by standard correction curves. Time-resolved measurements were recorded with a Horiba Jobin-Yvon IBH FL-322 Fluorolog 3 spectrometer by using the time-correlated single-photon counting (TCSPC) electronics FluoroHub. A pulsed laser diode LDH-P-C-373 (λ=373 nm, pulse duration 1.2 ns, max repetition rate 1MHz) and LDH-P-C-255 (λ=255 nm, pulse duration < 1.2 ns, max repetition rate 1MHz) was used to excite the sample and mounted directly on the sample chamber at 908. The photons were collected by using a TBX picosecond photon detection module single-photon-counting detector. The data was acquired by using the commercially available software DataStation (Horiba Jobin-Yvon) whereas data analysis was performed by using the commercially available software DAS56 (Horiba Jobin-Yvon). Quantum yield measurement were performed with Fluorolog-3 spectrofluorometer with a Quanta-φ integrating sphere as an accessory attached. The data was acquired by the commercially available software FluorEssence™ (Horiba Jobin-Yvon) version 3.5.

For all spectroscopic experiments, plastic cuvettes with a light path of 10 mm and dimensions of 10 × 10 mm from Brand with a spectroscopic cut-off at 220 nm were used for the water-based solvent, while quartz cuvettes were utilized for organic solvent, e.g. DMSO and HFP.

The pH titration measurements were carried out with a FiveEasy (Mettler Toledo) pH meter F20.

High-performance liquid chromatography (HPLC) experiments were performed on a LC-2000Plus HPLC system equipped with a UV-2075 detector and a Kromasil 100 C 18 5 μM LC column (250 × 4.6 mm, Agela) at a flow rate of 0.8 mL/min. The sample were dissolved in water at a concentration of 1.5 mg/mL.

2. pH titration experiment

2.1. pH titration photophysical experiment

The pH titration was conducted by using certified standard titrant solutions of 0.1 N NaOH, 1.0 N NaOH and 0.5 N H_2SO_4, which were drop-wise added to a solution of HINA, HPA or HAP (50 μM) in water. The pH value was acquired by a FiveEasy (Mettler Toledo) pH meter F20. After pH recording, absorbance and emission spectra were measured. The ground state pK_{a1} values were determined by an absorbance-based spectroscopic investigation of HINA, HPA and HAP in the pH range of 2.5-5.5 while the ground state pK_{a2} values were determined in the pH range of 5.5-10.

The absorbance (and emission) intensity at characteristic wavelength was plotted versus pH values of the solution. pK_a values were fitted using the literature equations:[1, 2]

$pK_{a1} = pH_x – \log (A_{H^+} - A_x) + \log (A_x – A_N)$ or $pK_{a1}{*}= pH_x – \log (I_{H^+} - I_x) + \log (I_x – I_N)$

$pK_{a2} = pH_x + \log (A_{B^-} - A_x) - \log (A_x – A_N)$ or $pK_{a2}{*}= pH_x + \log (I_{B^-} - I_x) - \log (I_x – I_N)$

Where A_{H^+}, A_x, A_N and A_{B^-} represent the absorbance of the protonated form, at the pH chosen, of the neutral form and of deprotonated form respectively. I_{H^+}, I_x, I_N and I_B represent the emission intensity of protonated form, at the pH chosen of the neutral form and of the deprotonated form respectively. The non-linear fitting was conducted with Origin 2018b software package.

The excited state (de)protonation behaviour was determined by emission-based spectroscopic investigation of HINA , HPA and HAP in the pH range of 5.5-10. The dyes were excited at an isobestic point, or at a wavelength were mainly the neutral form of the dye, or at a wavelength were mainly the anionic form of the dye is absorbing. Emission were recorded where only the anionic form emits. Excited-state acidities (pK_{a2}*) were estimated via the Förster cycle method (see further below).

2.1.1. Absorbance and emission spectra

2.1.1.1. 3-hydroxy-isonicotinic aldehyde (HINA)

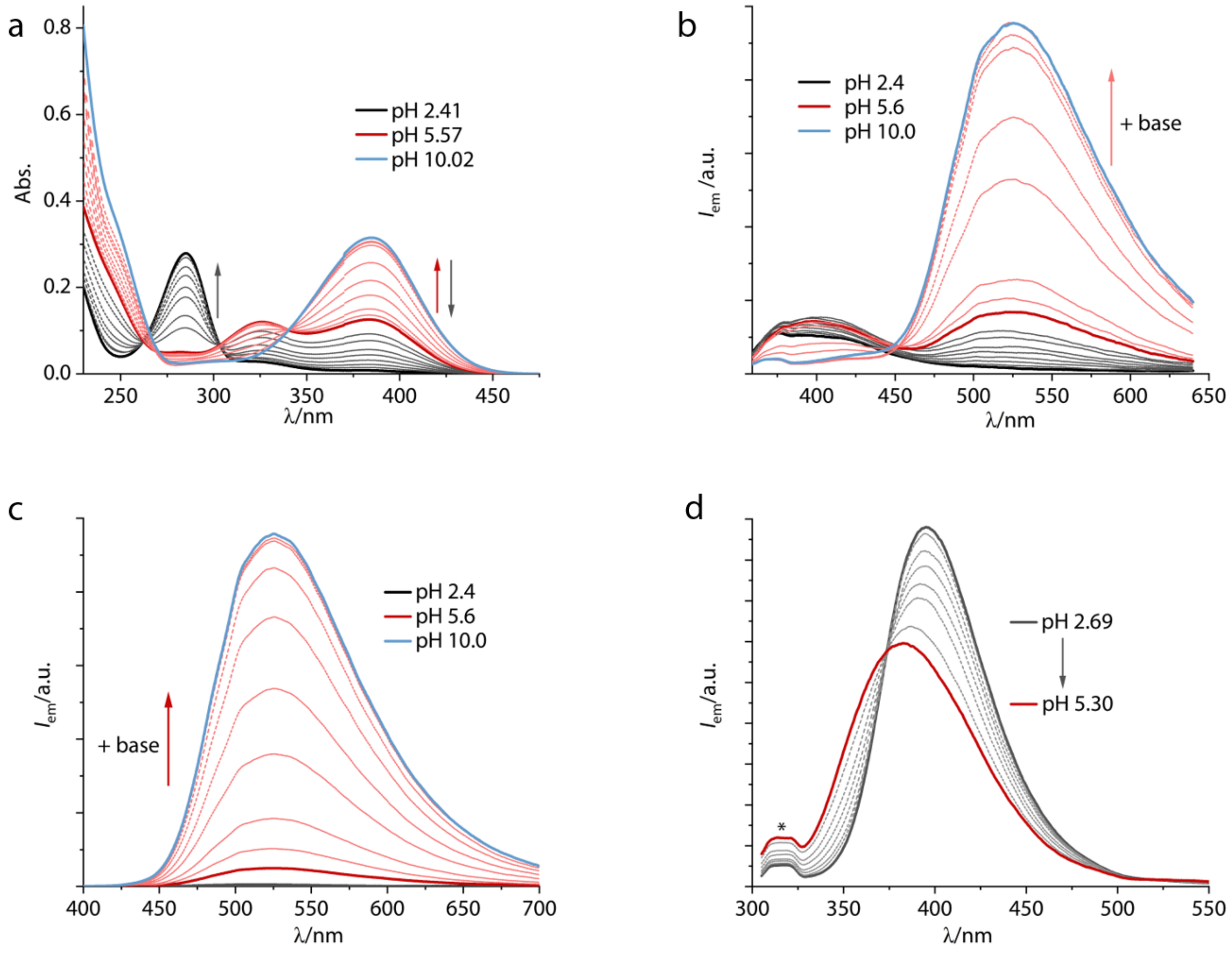

Figure S1. Absorbance (a) and emission (b-d) spectra of HINA (50 μM) titrated with a standard solution of H_2SO_4 (0.5 N, a series of black lines) and a standard solution of NaOH (0.1 N, a series of red lines) in MilliQ water at 25 °C. In b) λ_{ex} = 330 nm was used as the excitation wavelength while excitation was carried out in c) at 380 nm, and in d) at 285 nm. The asterisk in the emission spectrum indicates the Raman scattering band of water at the selected excitation wavelength.

2.1.1.2. 3-hydroxy-picolin aldehyde (HPA)

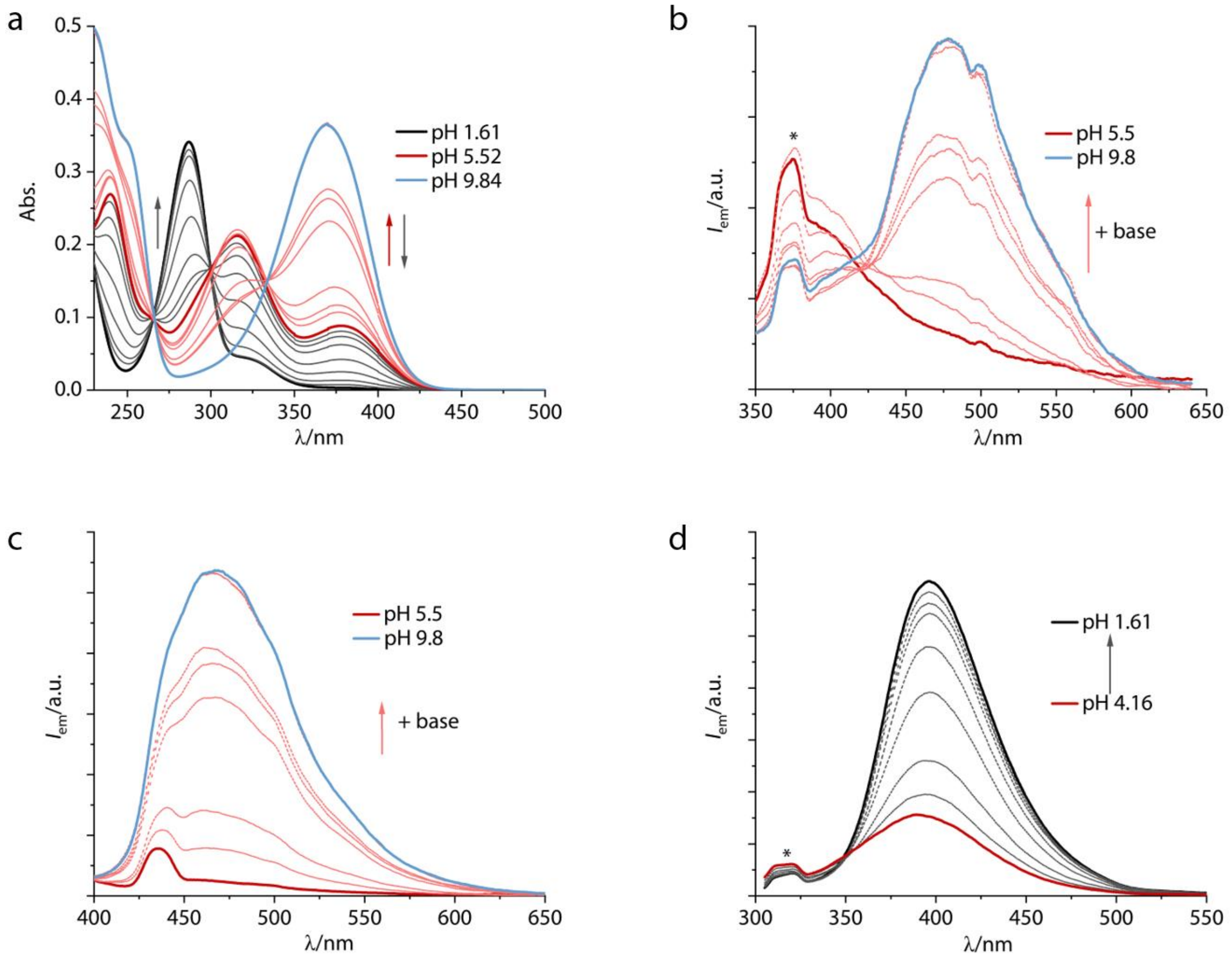

Figure S2. Absorbance (a) and emission (b-d) spectra of HPA (50 μM) titrated with a standard solution of H_2SO_4 (0.5 N, a series of black lines) and a standard solution of NaOH (0.1 N, a series of red lines) in MilliQ water at 25 °C. In b) λ_{ex} = 330 nm was used as the excitation wavelength while excitation was carried out in c) at 380 nm, and in d) at 285 nm. The asterisk in the emission spectrum indicates the Raman scattering band of water at the selected excitation wavelength.

2.1.1.3. 3-hydroxy-4-acetyl pyridine (HAP)

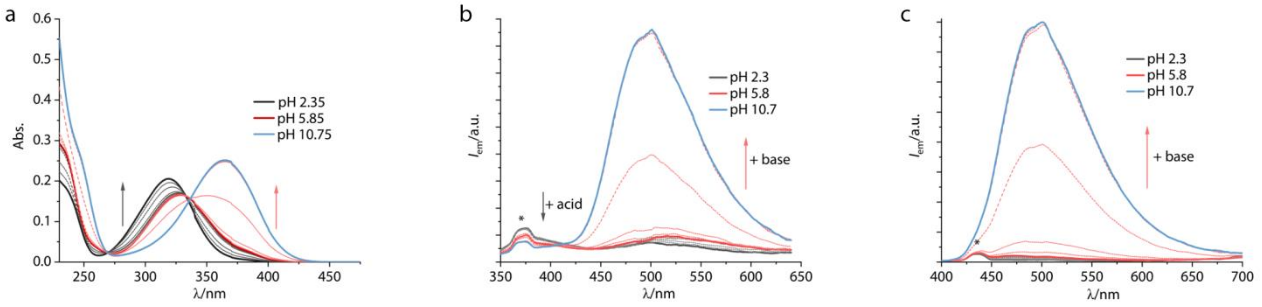

Figure S3. Absorbance (a) and emission (b-c) spectra of HAP (50 μM) titrated with a standard solution of H_2SO_4 (0.5 N, a series of black lines) and a standard solution of NaOH (0.1 N, a series of red lines) in MilliQ water at 25 °C. In b) λ_{ex} = 330 nm was used as the excitation wavelength while excitation was carried out in c) at 380 nm. The asterisk in the emission spectrum indicates the Raman scattering band of water at the selected excitation wavelength.

2.1.2. pK_a fitting

2.1.2.1. 3-hydroxy-isonicotinic aldehyde (HINA)

Table S1.pK_{a1} value of HINA determined by absorbance titration The emission-based fitting value is shown for comparsion.

	absorbance ($\lambda_{abs.}$ = 286 nm)	emission ($\lambda_{ex.}$ = 286 nm, $\lambda_{em.}$ = 397 nm)
pK_{a1}	3.97 ± 0.05	3.88 ± 0.08

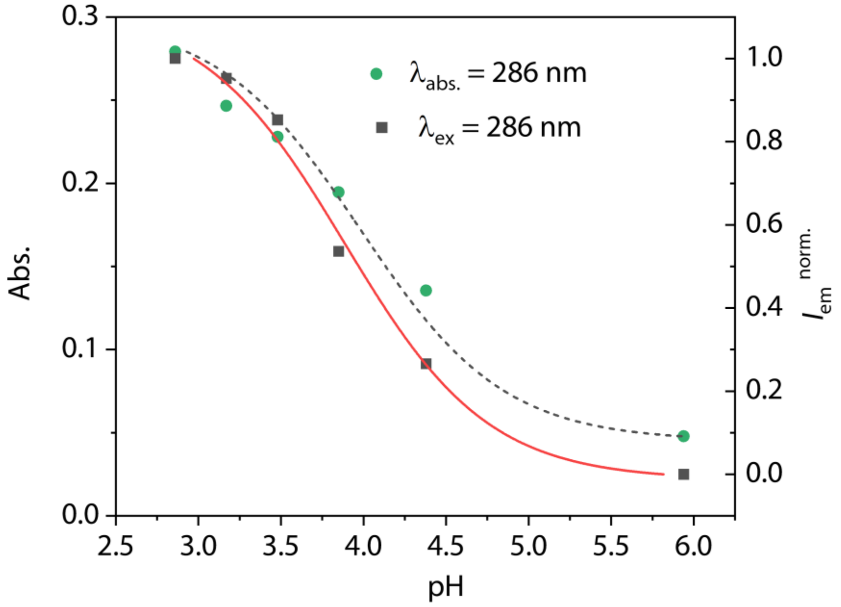

Figure S4. Plot of absorbance ($\lambda_{abs.}$ = 286 nm, green dots) and [0,1]-normalized emission ($\lambda_{ex.}$ = 286 nm, $\lambda_{em.}$ = 397 nm, black square) intensity change of HINA (50 μM) with pH values in the range of 2.56-5.94. Fits are shown as dashed black line (absorbance) and solid red line (emission), the fitted pK_{a1} values are shown in the Table S1.

Table S2. pK_{a2} value of HINA determined by absorbance titration. For comparison, fitting values are given for emission-based titrations where selectively the neutral form, both neutral and anionic form (isosbestic point) and only the anionic form of the fluorophore where excited, respectively. The closely maching values suggest that deprotonation of the fluorophore does not occur during the lifetime of the excited state.

	absorbance	emission		
	$\lambda_{abs.}$ = 385 nm	λ_{ex} = 325 nm	λ_{ex} = 340 nm	λ_{ex} = 385 nm
pK_{a2}	7.05 ± 0.01	7.10 ± 0.01	7.08 ± 0.01	6.98 ± 0.01

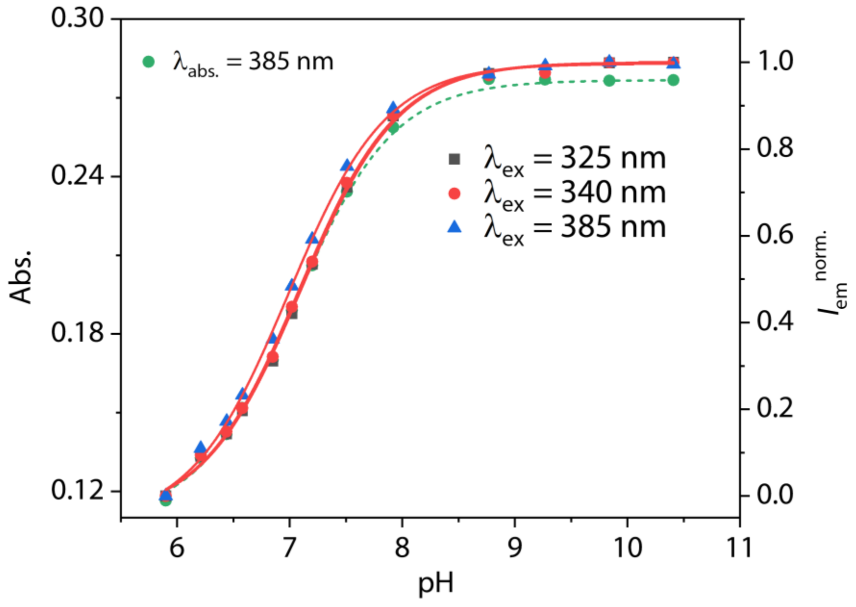

Figure S5. Plot of absorbance ($\lambda_{abs.}$ = 385 nm, green dots) and [0,1]-normalized emission ($\lambda_{ex.}$ = 325 nm, black squares, $\lambda_{ex.}$ = 340 nm, red dots; $\lambda_{ex.}$ = 385 nm, blue triangels; $\lambda_{em.}$ = 397 nm,) intensity change of HINA (50 µM) with pH values in the range of 5.90-10.41 Fits are shown as dashed green line (absorbance) and solid red lines (emission), the fitted pK_{a2} values are showed in the Table S2. ***Notes***: the excitation wavelengths used here are characteristic for the neutral form (325 nm), isosbestic point of neutral form and depronated form (340 nm) and the depronated form (385 nm), respectively.

2.1.2.2. 3-hydroxy-picolin aldehyde (HPA)

Table S3. pK_{a1} value of HPA determined by absorbance titration. The emission-based fitting value is shown for comparsion.

	absorbance ($\lambda_{abs.}$ = 286 nm)	emission ($\lambda_{ex.}$ = 286 nm, $\lambda_{em.}$ = 397 nm)
pK_{a1}	3.43 ± 0.02	3.53 ± 0.00

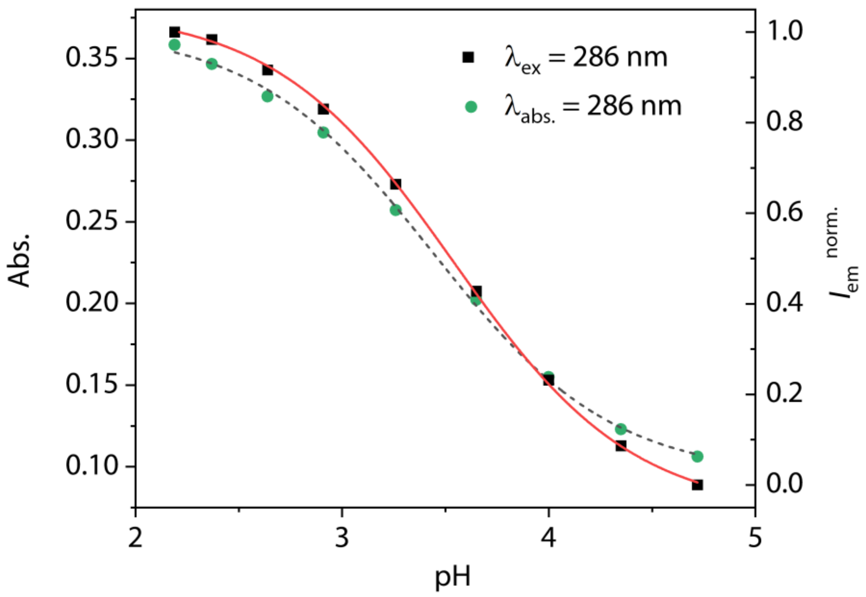

Figure S6. Plot of absorbance ($\lambda_{abs.}$ = 286 nm, green dots) and [0,1]-normalized emission ($\lambda_{ex.}$ = 286 nm, $\lambda_{em.}$ = 397 nm, black square) intensity change of HPA (50 µM) with pH values in the range of 2.19-4.72. Fits are shown as dashed black line (absorbance) and solid red line (emission), the fitted pK_{a1} values are showed in the Table S3.

Table S4. pK_{a2} value of HPA determined by absorbance titration. For comparison, fitting values are given for emission-based titrations where selectively the neutral form, both neutral and anionic form (isosbestic point) and only the anionic form of the fluorophore where excited, respectively. The closely maching values suggest that deprotonation of the fluorophore does not occur during the lifetime of the excited state.

	absorbance titration $\lambda_{abs.}$ = 369 nm	emission λ_{ex} = 313 nm	λ_{ex} = 330 nm	λ_{ex} = 369 nm
pK_{a2}	7.21 ± 0.04	7.23 ± 0.02	7.19 ± 0.03	7.05 ± 0.03

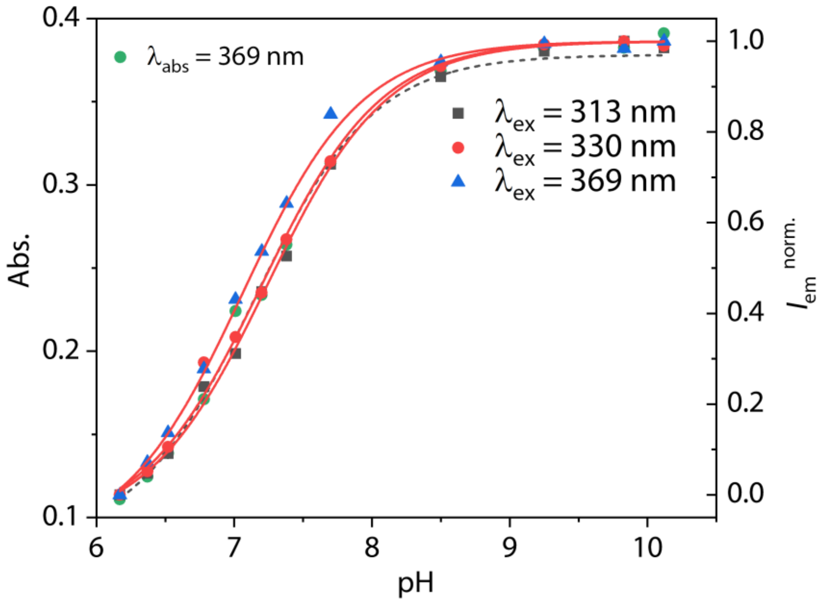

Figure S7. Plot of absorbance ($\lambda_{abs.}$ = 369 nm, green dots) and [0,1]-normalized emission ($\lambda_{ex.}$ = 313 nm, black squares, $\lambda_{ex.}$ = 330 nm, red dots; $\lambda_{ex.}$ = 369 nm, blue triangels; $\lambda_{em.}$ = 467 nm,) intensity change of HPA (50 µM) with pH values in the range of 6.17-10.12, Fits are shown as dashed black line (absorbance) and solid red lines (emission), the fitted pK_{a2} values are showed in the Table S4. ***Notes***: the excitation wavelengths used here are characteristic for the neutral form (313 nm), isosbestic point of neutral form and depronated form (330 nm) and the depronated form (369 nm), respectively.

2.1.2.3. 3-hydroxy-4-acetyl pyridine (HAP)

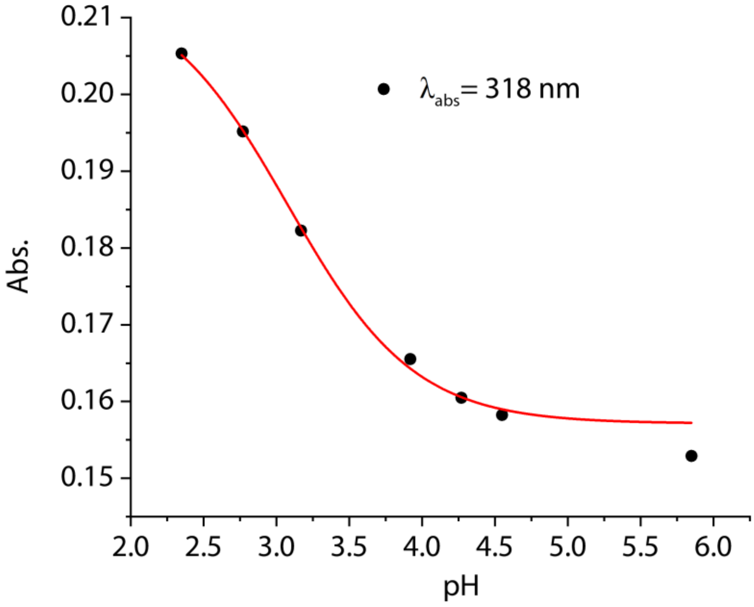

Figure S8. Plot of absorbance ($\lambda_{abs.}$ = 318 nm, black dots) intensity at change of HAP (50 µM) with pH values in the range of 2.3-5.8, the fits shown as red line, pK_{a1} = 3.07 ± 0.03.

Table S5. pK_{a2} value of HAP determined by absorbance titration. For comparison, fitting values are given for emission-based titrations where selectively the neutral form, both neutral and anionic form (isosbestic point) and only the anionic form of the fluorophore where excited, respectively. The closely maching values suggest that deprotonation of the fluorophore does not occur during the lifetime of the excited state.

	absorbance titration $\lambda_{abs.}$ = 365 nm	emission λ_{ex} = 326 nm	λ_{ex} = 337 nm	λ_{ex} = 365 nm
pK_{a2}	8.13 ± 0.02	8.18 ± 0.01	8.12 ± 0.01	8.03 ± 0.01

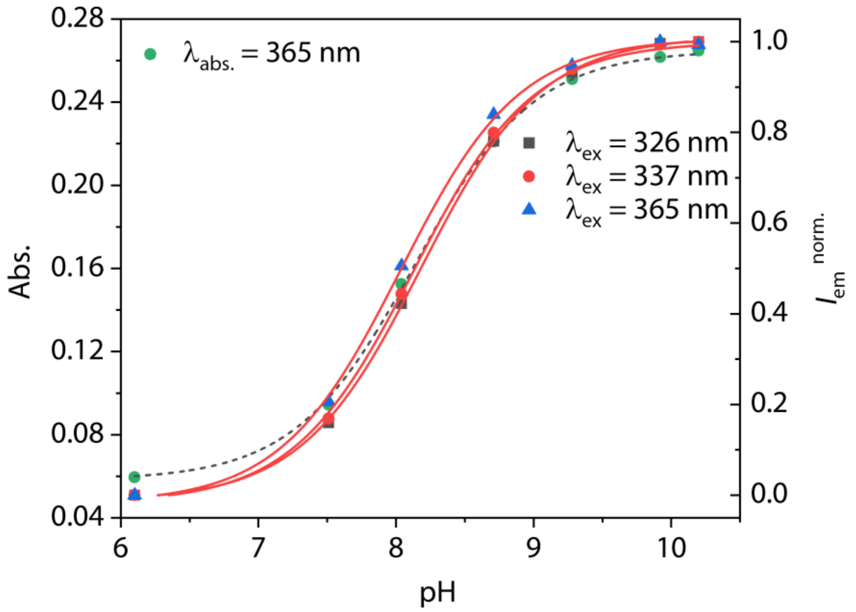

Figure S9. Plot of absorbance ($\lambda_{abs.}$ = 365 nm, green dots) and [0,1]-normalized emission ($\lambda_{ex.}$ = 326 nm, black squares, $\lambda_{ex.}$ = 337 nm, red dots; $\lambda_{ex.}$ = 366 nm, blue triangels; $\lambda_{em.}$ = 486 nm,) intensity change of HAP (50 µM) with pH values in the range of 6.10-9.92, the fits shown as black dash line (absorbance) and red line (emission), the pK_{a2} values are showed in the Table S5. ***Notes***: the excitation wavelengths used here are characteristic for the neutral form (326 nm), isosbestic point of neutral form and depronated form (337 nm) and the depronated form (365 nm), respectively.

2.2. pK_a value determination by pH meter

The pH titration was conducted by using 1.0 N NaOH and 0.5 N H_2SO_4 as titrant solution, which was drop wisely added to a solution of HINA, HPA and HAP (10 mM) in water. The pH value was acquired by a FiveEasy (Mettler Toledo) pH meter F20. The data was processed with a pH-fitting software package available online.[3, 4]

2.2.1. 3-hydroxy-isonicotinic aldehyde (HINA)

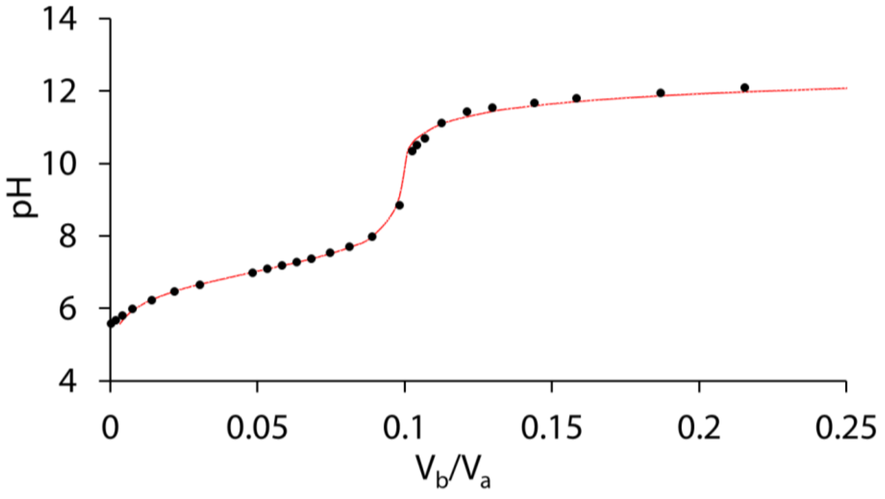

Figure S10. pH titration curve of HINA (10 mM) that was titrated with a standard solution of NaOH (0.1 N) in MilliQ water, the red dash line is the fitting curve, pK_{a2} = 7.02.

2.2.2. 3-hydroxy-picolin aldehyde (HPA)

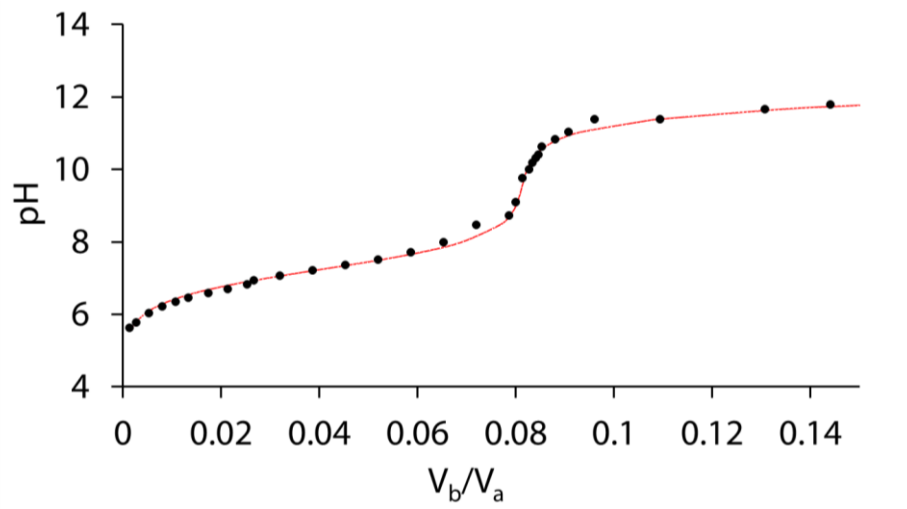

Figure S11. pH titration curve of HPA (10 mM) that was titrated with a standard solution of NaOH (0.1 N) in MilliQ water, the red dash line is the fitting curve, pK_{a2} = 7.24.

2.2.3. 3-hydroxy-4-acetyl pyridine (HAP)

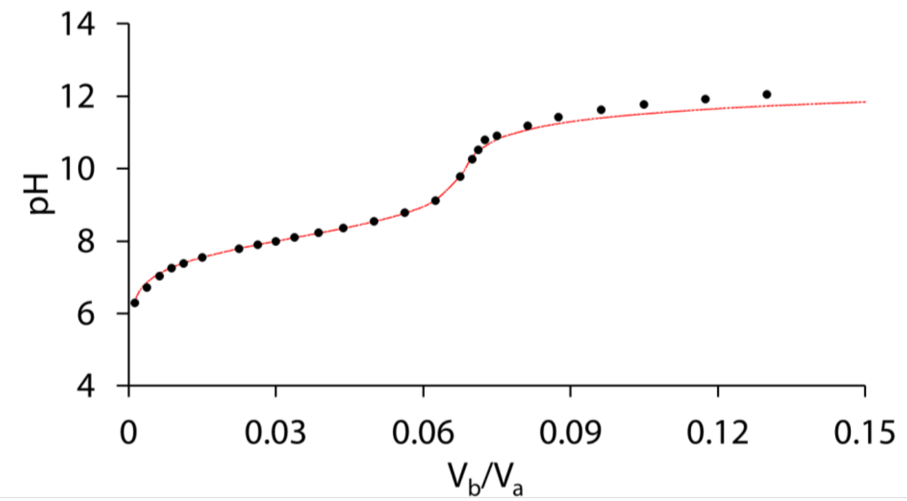

Figure S12. pH titration curve of HAP (10 mM) that was titrated with a standard solution of NaOH (0.1 N) in MilliQ water, the red dash line is the fitting curve, pK_{a2} = 8.10.

Table S6. The comparation of pK_a values were determined with pH titration, absorbance spectra and emission spectra.

	pK_{a1}			pK_{a2}		
	pH meter	absorbance	emission	pH meter	absorbance	emission
HINA	3.54	3.97 ± 0.05	3.88 ± 0.08	7.02	7.05 ± 0.01	6.98 ± 0.01
HPA	3.11	3.43 ± 0.02	3.53 ± 0.00	7.24	7.14 ± 0.04	7.05 ± 0.03
HAP	2.58	3.07 ± 0.03	-	8.10	8.03 ± 0.01	8.13 ± 0.02

Notes: pK_a values from emission-based titrationwere obtained by the excitation of the protonated (pK_{a1}) or deprotonated (pK_{a2}) form of the fluorophore, and recording of the corresponding emission from the protonated (pK_{a1}) or deprotonated (pK_{a2}) species. Thus, these emission-based pK_a values resemeble ground.state behaviour.

2.3. Calculation of excited-state acidities (pK_{a2}*) of HINA, HPA and HAP using Förster cycle analysis

The “thermodynamic”acidities (pK_{a2}^*) of the excited state of HINA, HPA and HAP were estimated from the experminentally available absorbance and emission maxima energies (Table 1 in the main text, of the protonated and deprotonated species after conversion into cm^{-1} units, and the ground-state pK_a value obtained from absorbance- and pH-meter based titrations (Table 1 in the main text) through use of the literature equations[5]:

$$pK_a^* = pK_a - \frac{h\nu_1 - h\nu_2}{2.3\,RT}$$

$$\tilde{\nu}_{00}^{\mathrm{A-}} = \frac{\tilde{\nu}_{\mathrm{A-}}^{a} + \tilde{\nu}_{\mathrm{A-}}^{f}}{2}$$

$$\tilde{\nu}_{00}^{\mathrm{HA}} = \frac{\tilde{\nu}_{\mathrm{HA}}^{a} + \tilde{\nu}_{\mathrm{HA}}^{f}}{2}$$

$$\Delta pK_a = \frac{\mathrm{Nhc}}{2.3RT}\left(\tilde{\nu}_{00}^{\mathrm{A-}} - \tilde{\nu}_{00}^{\mathrm{HA}}\right) = \frac{0.625}{T}\left(\tilde{\nu}_{00}^{\mathrm{A-}} - \tilde{\nu}_{00}^{\mathrm{HA}}\right)$$

The parameters $\tilde{\nu}_{\mathrm{A-}}^{a}$ and $\tilde{\nu}_{\mathrm{A-}}^{f}$ denotate the maxima of the absorbance and fluorescence spectra of the anion. Likewise, $\tilde{\nu}_{\mathrm{AH}}^{a}$ and $\tilde{\nu}_{\mathrm{AH}}^{f}$ denotate the maxima of the absorbance and fluorescence spectra of the neutral form of HINA, HPA and HAP. Under these approximations, the excited-state acidities (pK_{a2}^*) of HINA, HPA and HAP were calculated to reach −5.5, −3.7 and 1.13, respectively. These acidities are so strong that excitation of the neutral form of HINA, HPA and HAP should lead to a complete deprotonation of the fluorophores in water and one would expect to witness only the emission of the anionic form, ***if*** the deprotonation occurs within the lifetime of the excited state. Clearly, this is not the case because the absorbance- and fluorescence-based pH-titration plots essentially overlay (see Figure 1d in the main text and Figures S5, S7 and S9), suggesting that deprotonation of HINA, HPA and HAP does not occur upon excitation of the neutral forms of the fluorophores. We believe that a fast intramolecular ESIPT process between the adjacent HO- and CHO- moieties (see Figure S73) intercepts proton transfer to the solvent, and thereby preven the expected fluorescence behaviour based on the calculated pK_a* values. [Similar deviations between predicted pK_a* values and experimental observations have been described in the literature for systems that do not deprotonate within the lifetime of the excited state.[6] For hydroxarenes, this behaviour is unusual; unlike HINA, HPA and HAP, many reported hydroxarenes are indeed strong photoacids.[5, 6]

3. Absorbance and emission spectra in organic solvents and solvent mixtures

Table S7. Information of solvent was used in the mixed media measurement

Solvent	Supplier/Info	Water content /m %	Water content /mM
Dry ACN	Sigma Aldrich	0.005	2.1
Dry DMSO	Across Organic	< 0.005	< 3.0

3.1. 3-hydroxy-isonicotinic aldehyde (HINA)

3.1.1. Spectra of HINA in acetonitrile (ACN) upon addition of water.

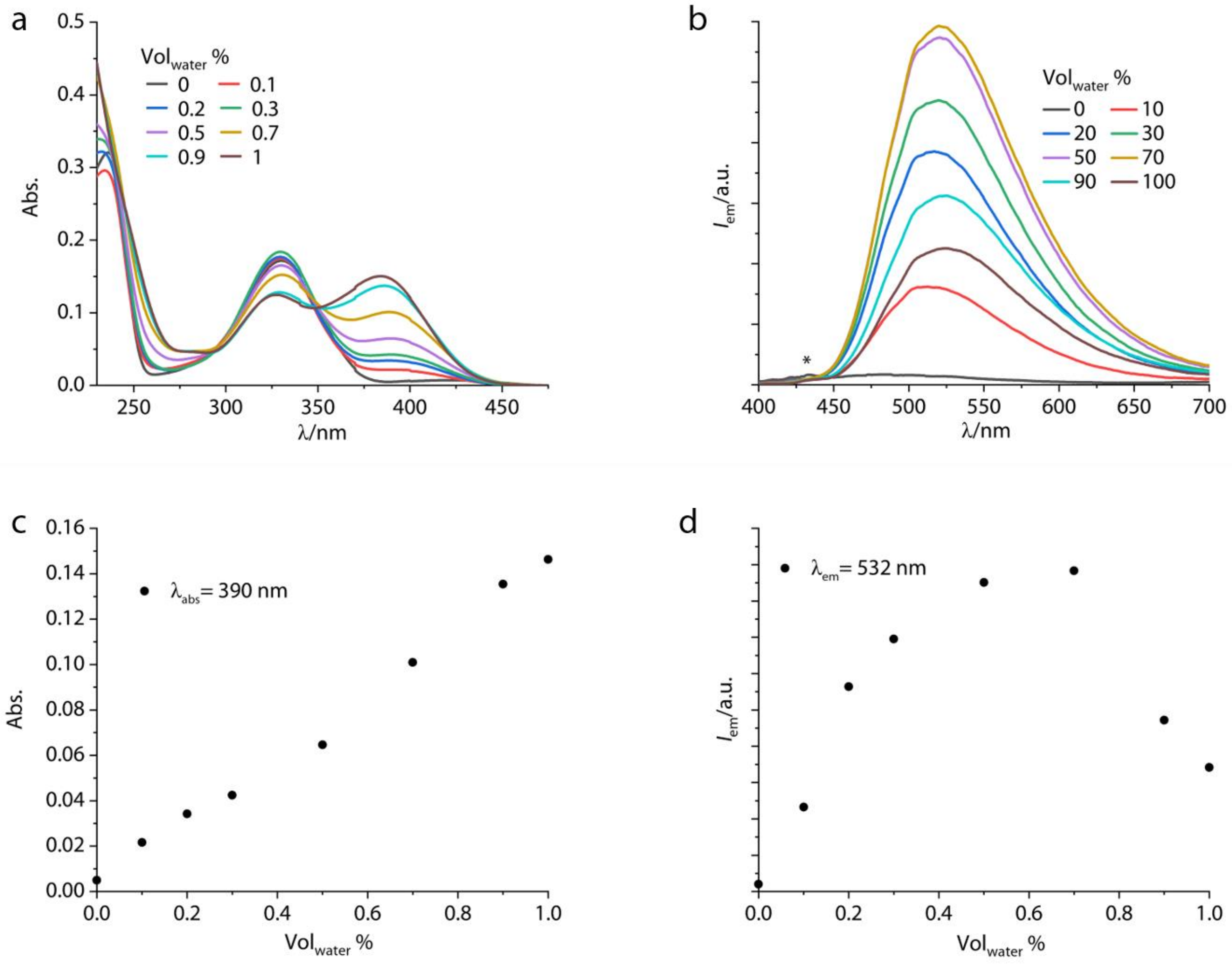

Figure S13. Absorbance (a) and emission (b, λ_{ex} = 380 nm) spectra of HINA (50 μM) in a series of mixture solvent of ACN and water (ACN/water, v/v) at 25 °C. c) Plot of the absorbance intensity at 390 nm as a function of the water content in the solvent mixture. d) Plot of the emission intensity at 532 nm as a function of the water content in the solvent mixture. The asterisk in the emission spectrum indicates the Raman scattering band of water at the selected excitation wavelength.

3.1.2. Spectra of HINA in dimethyl sulfoxide (DMSO) upon addition of water

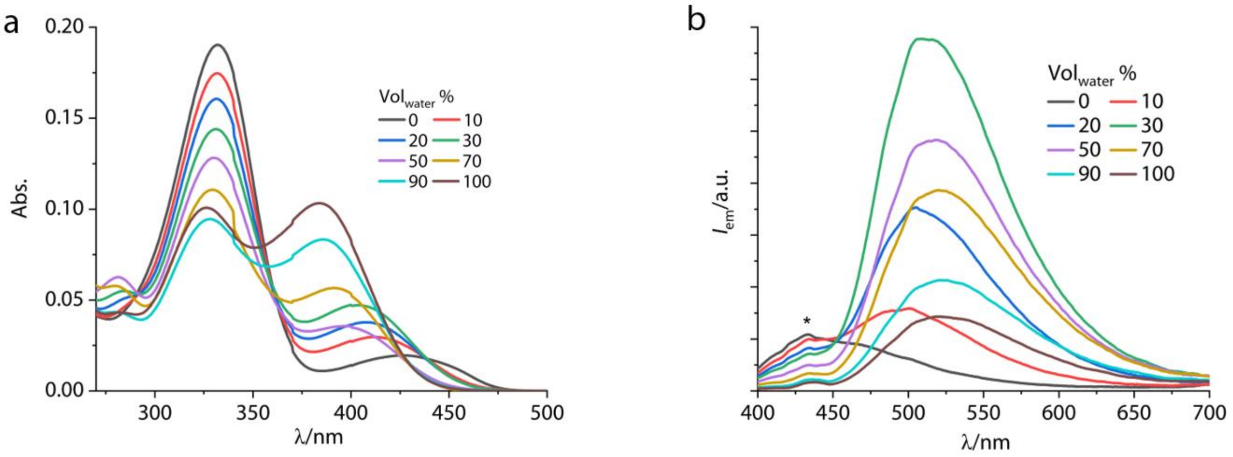

Figure S14. Absorbance (a) and emission (b, λ_{ex} = 380 nm) spectra of HINA (50 μM) in a series of mixture solvent of DMSO and water (DMSO/water, v/v) at 25°C. The asterisk in the emission spectrum indicates the Raman scattering band of water at the selected excitation wavelength.

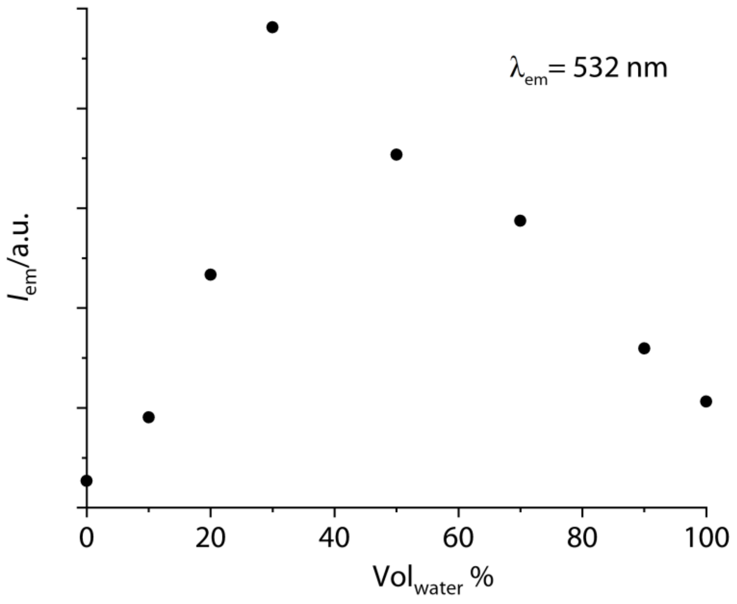

Figure S15. Plot of the emission intensity at 532 nm as a function of the water content in the solvent mixture.

3.1.3. Spectra of HINA in DMSO upon addition of anhydrous base or urea

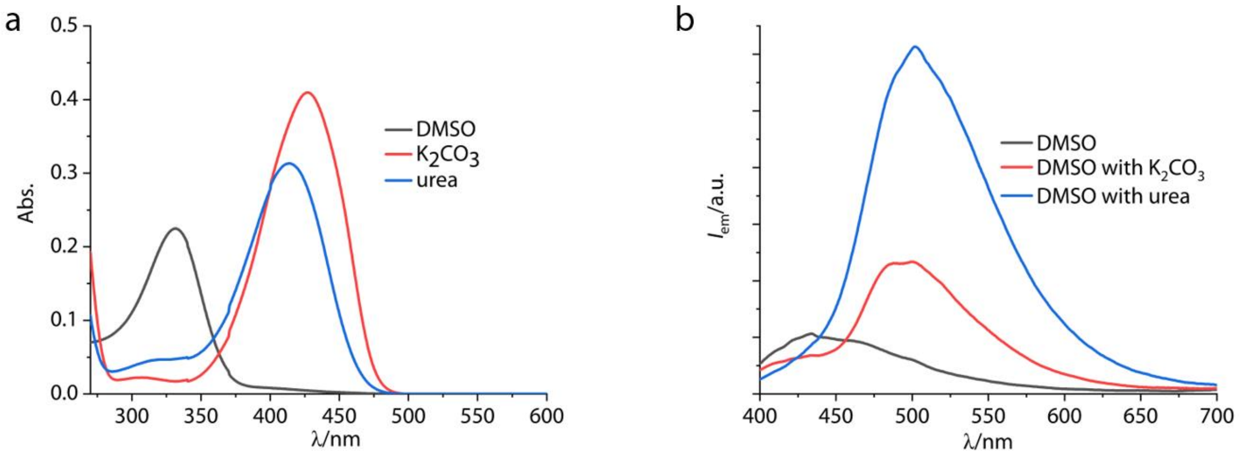

Figure S16. Absorbance (a) and emission (b, λ_{ex} = 380 nm) spectra of HINA (50 μM) in DMSO (black line) and with K_2CO_3 (0.3 M, red line) and urea (0.3 M, blue line) addition.

3.1.4. Spectra of HINA in MeOH upon addition of anhydrous base

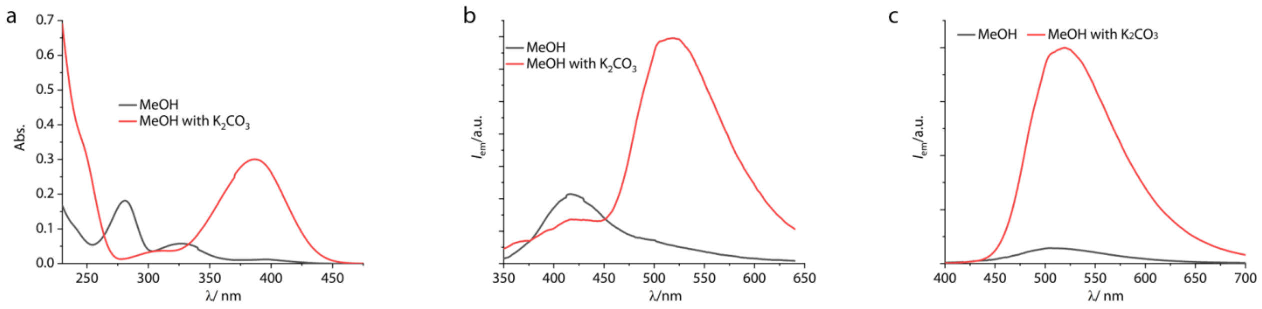

Figure S17. Absorbance (a) and emission (b-c) spectra of HINA (50 μM) in MeOH and with K_2CO_3 (0.3 M) at 25 °C. In b) λ_{ex} = 330 nm was used as the excitation wavelength while excitation was carried out in c) at 380 nm.

3.1.5. Spectra of HINA in ethylene glycol (EG) upon addition of anhydrous base

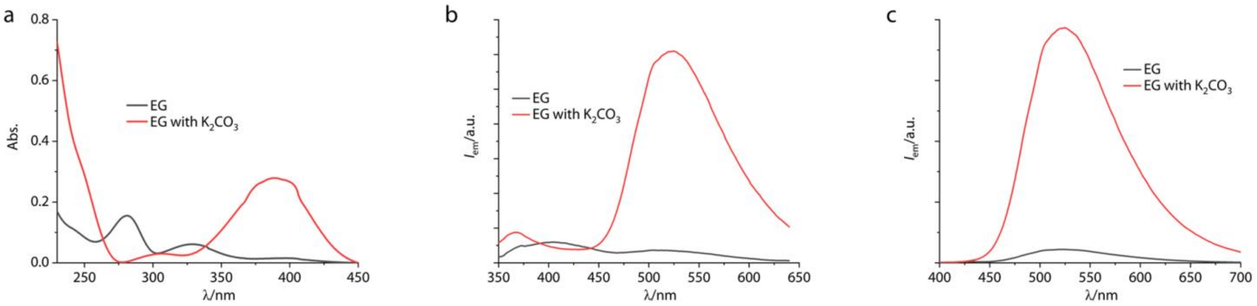

Figure S18. Absorbance (a) and emission (b-c) spectra of HINA (50 μM) in EG and with K_2CO_3 (0.3 M) addition at 25 °C. In b) λ_{ex} = 330 nm was used the excitation wavelength while excitation was carried out in c) at 380 nm.

3.1.6. Spectra of HINA in hexafluoro-2-propanol (HFP)

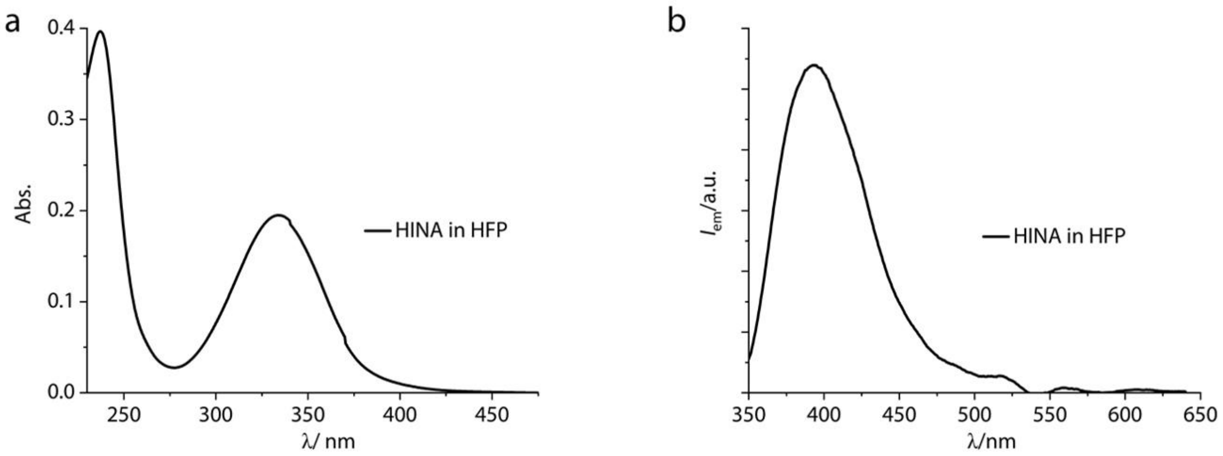

Figure S19. Absorbance (a) and emission (b, λ_{ex} = 330 nm) spectrum of HINA (50 μM) in HFP at 25 °C.

Note: HFP is so acidic that can’t support to form anion of HINA.

3.2. 3-hydroxy-picolin aldehyde (HPA)

3.2.1. Spectra of HPA in ACN upon addition of water

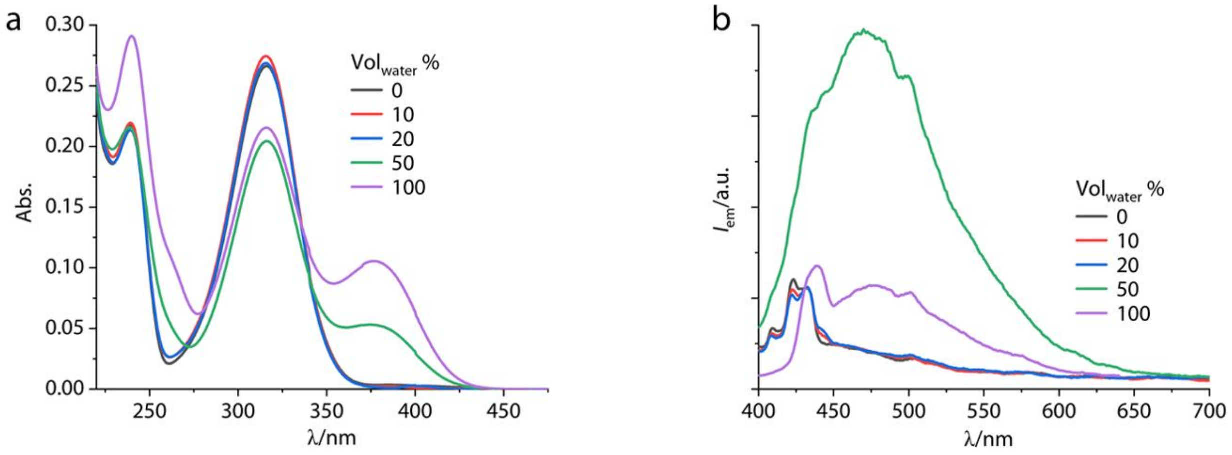

Figure S20. Absorbance (a) and emission (b) spectra of HPA (50 μM, λ_{ex} = 380 nm) in ACN-water mixtures (ACN/water, v/v) at 25 °C.

3.2.2. Spectra of HPA in MeOH upon addition of anhydrous base

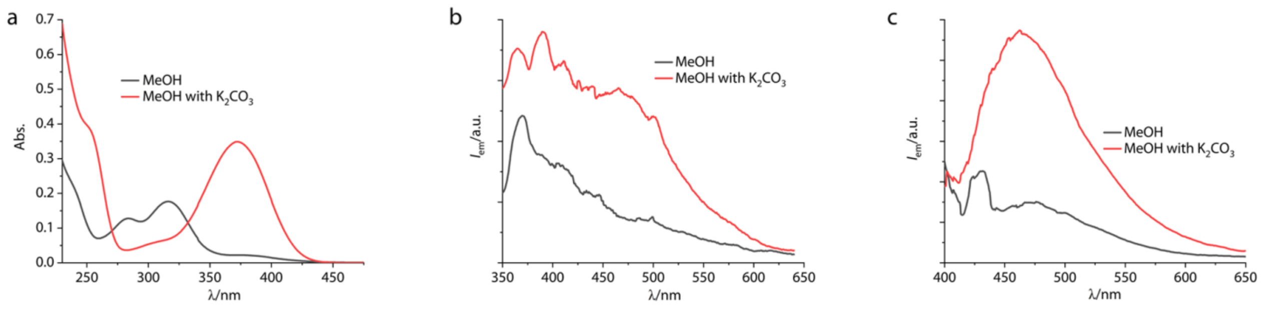

Figure S21. Absorbance (a) and emission (b-c) spectra of HPA (50 μM) in MeOH with K_2CO_3 (0.3 M) addition at 25 °C. In b) λ_{ex} = 330 nm was used as the excitation wavelength while excitation was carried out in c) at 380 nm.

3.2.3. Spectra of HPA in EG upon addition of anhydrous base

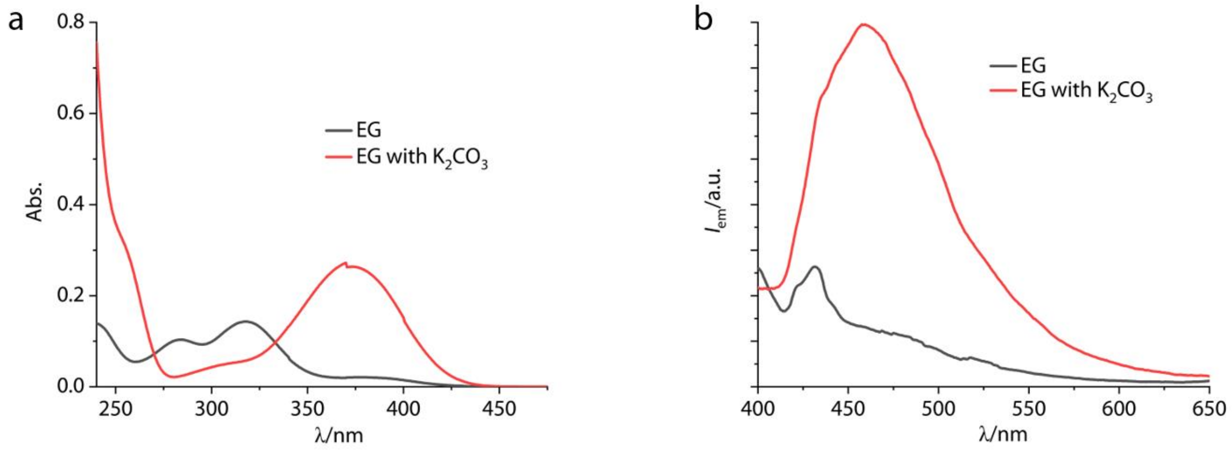

Figure S22. Absorbance (a) and emission (b, λ_{ex} = 380) spectra of HPA (50 μM) in EG and with K_2CO_3 (0.3 M) addition at 25 °C.

3.3. 3-hydroxy-4-acetyl pyridine (HAP)

3.3.1. Spectra of HAP in ACN upon addition of water

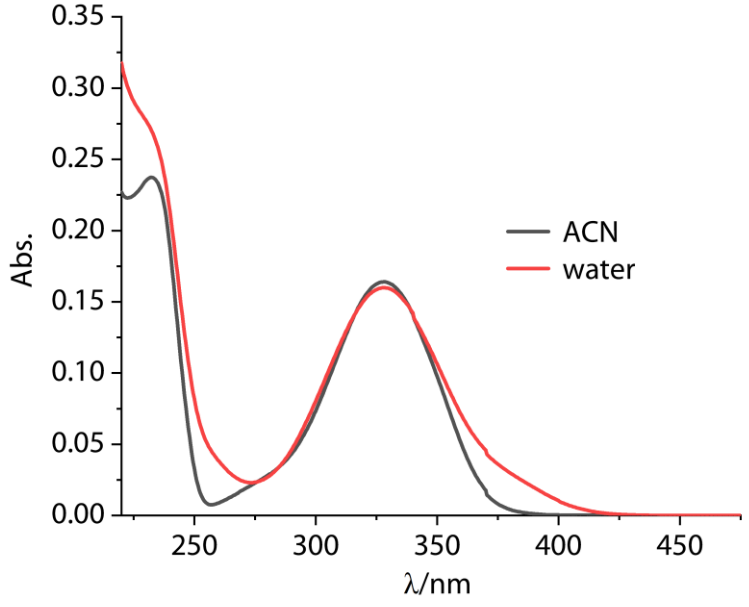

Figure S23. Absorbance spectra of HAP (50 μM) in ACN-water mixtures at 25 °C.

3.3.2. Spectra of HAP in DMSO upon addition of anhydrous base

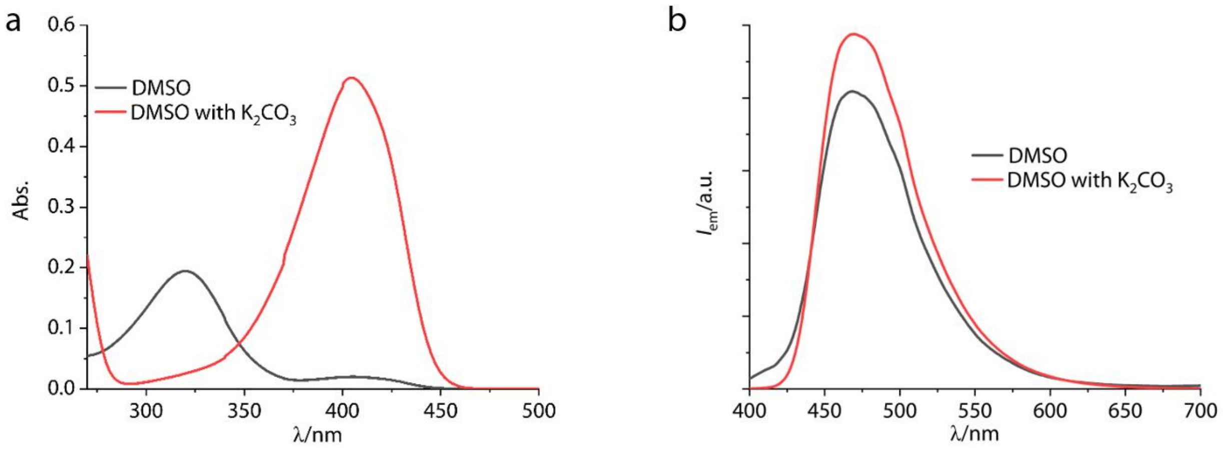

Figure S24. Absorbance (a) and emission (b, λ_{ex} = 380 nm) spectra of HAP (50 μM) in DMSO and with K_2CO_3 (0.3 M, red line) at 25 °C .

3.3.3. Spectra of HAP in MeOH upon addition of anhydrous base

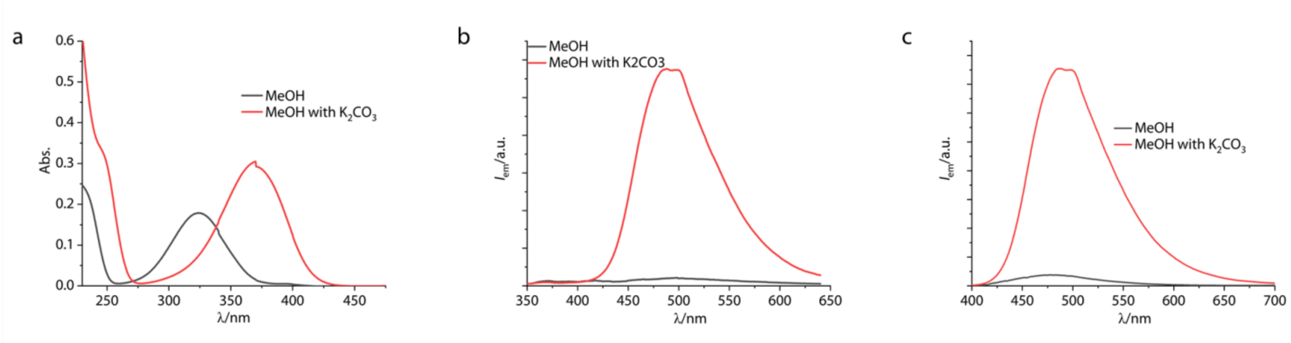

Figure S25. Absorbance (a) and emission (b-c) spectra of HAP (50 μM) in MeOH with K_2CO_3 (0.3 M) addition at 25 ℃. In b) λ_{ex} = 330 nm was used as the excitation wavelength while excitation in c) was carried out at 380 nm.

3.3.4. Spectra of HAP in EG upon addition of anhydrous base

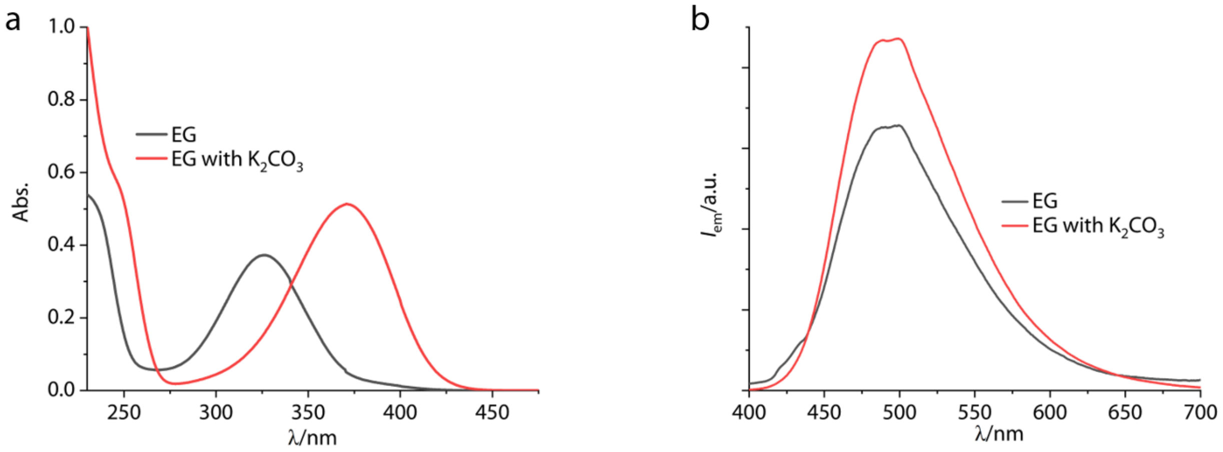

Figure S26. Absorbance (a) and emission (b, λ_{ex} = 380 nm) spectra of HAP (50 μM) in EG and with K_2CO_3 (0.3 M) addition at 25 °C.

4. Emission quantum yields in organic solvents

Table S8. Emission quantum yield of HINA in absence and presence of anhydrous base (K_2CO_3) in selected organic solvent.

	Acetonitrile	DMSO	Methanol
In absence of base	0.2 %	2.1 %	4.7 %
In presence of base	0.3 %	2.9 %	24 %

5. Concentration-depencency investigations

5.1. Concentration increase of HINA in buffer

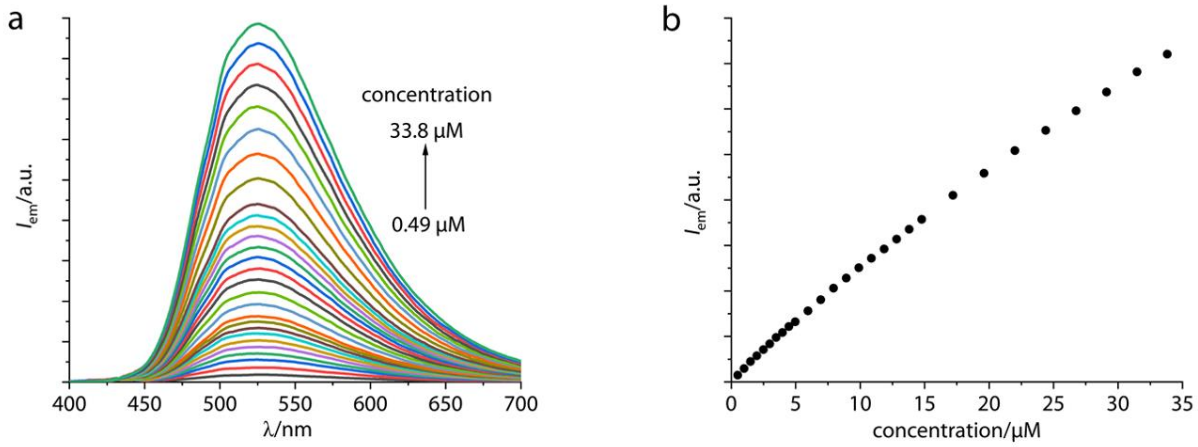

Figure S27. a) Emission spectra of HINA (λ_{ex} = 380 nm) in bicarbonate buffer (25 mM) at pH 9.5 and 25 °C. The concentration of HINA was increased from 0.49 μM to 33.8 μM. b) Plot of emission intensity at 533 nm with the concentration of HINA.

5.2. Concentration decrease of HINA in buffer

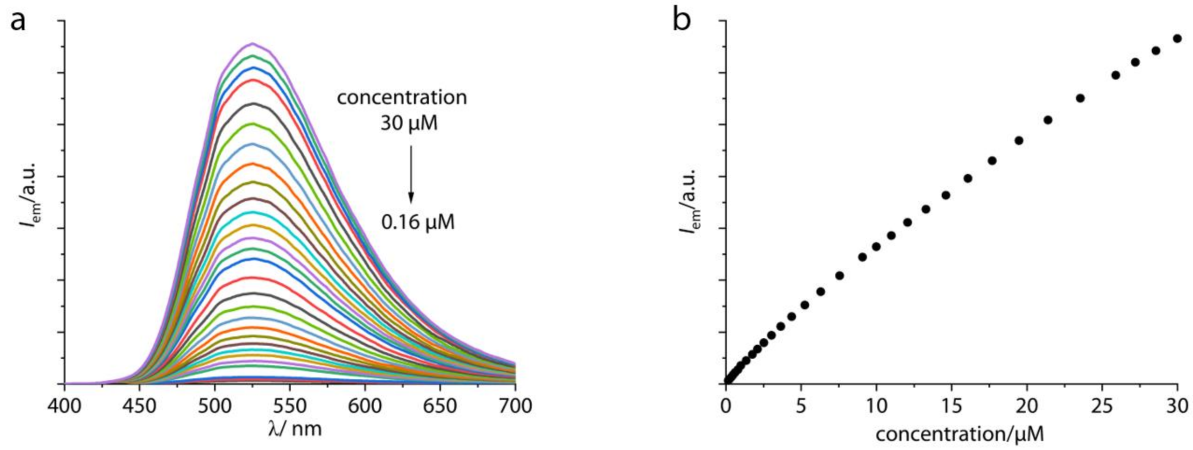

Figure S28. a) Emission spectra of HINA (λ_{ex} = 380 nm) in bicarbonate buffer (25 mM) at pH 9.5 and 25 °C. The concentration of HINA was decreased from 30 μM to 0.16 μM; b) Plot of emission intensity at 533 nm with the concentration of HINA.

Note: Contrary to the presented HINA dye, aggregation events of pyrenes[7] and synthetic GFP chromophores[8] occur in the concentration range around or below 10 μM.

6. Salicyladehyde (SA) measurement

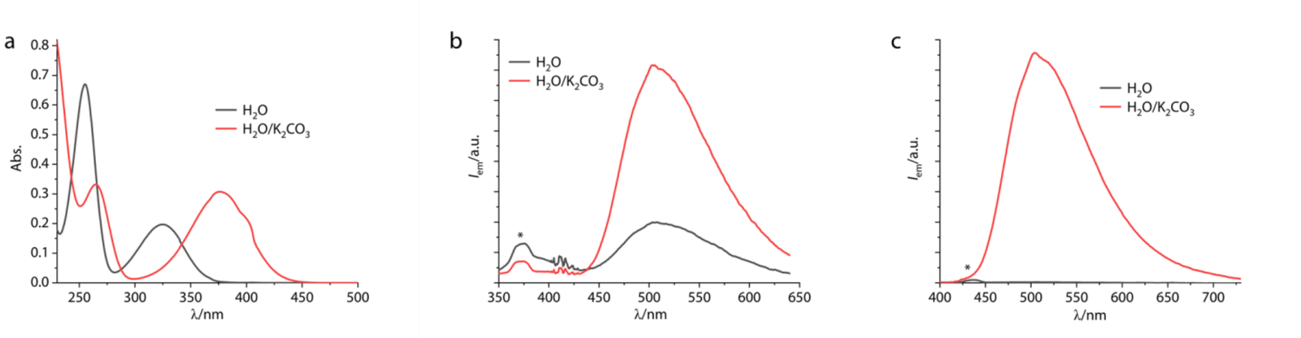

Figure S29. Absorbance (a) and emission (b-c) spectra of SA (50 µM) in MilliQ water and with K_2CO_3 (0.3 M) addition at 25 °C. In b) λ_{ex} = 330 nm was used the excitation wavelength while excitation was carried out in c) at 380 nm. The asterisk in the emission spectrum indicates the Raman scattering band of water at the selected excitation wavelength.

7. Pyridoxal measurement

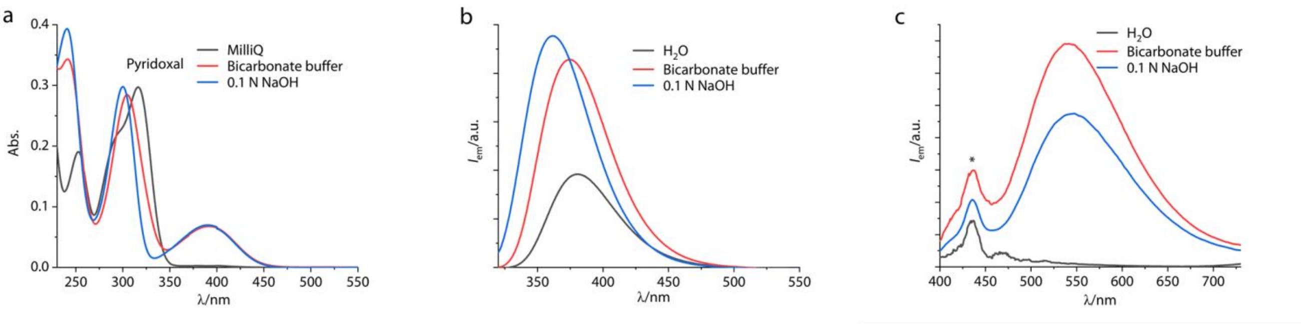

Figure S30. Absorbance (a) and emission (b-c) spectra of pyridoxal (50 µM) in MilliQ water, bicarbonate buffer (pH = 9.5) and with NaOH (0.1 N) at 25 °C. In b) λ_{ex} = 300 nm was used the excitation wavelength while excitation was carried out in c) at 390 nm. Pyridoxal has a strong blue emission while its green emission is very weak. Thus, measurement results in b) were obtained with a medium detector sensitivity of the fluorimeter, while a high detector sensitivity was used in c). The asterisk in the emission spectrum indicates the Raman scattering band of water at the selected excitation wavelength.

8. Purity determination of HINA by analytical HPLC measurements

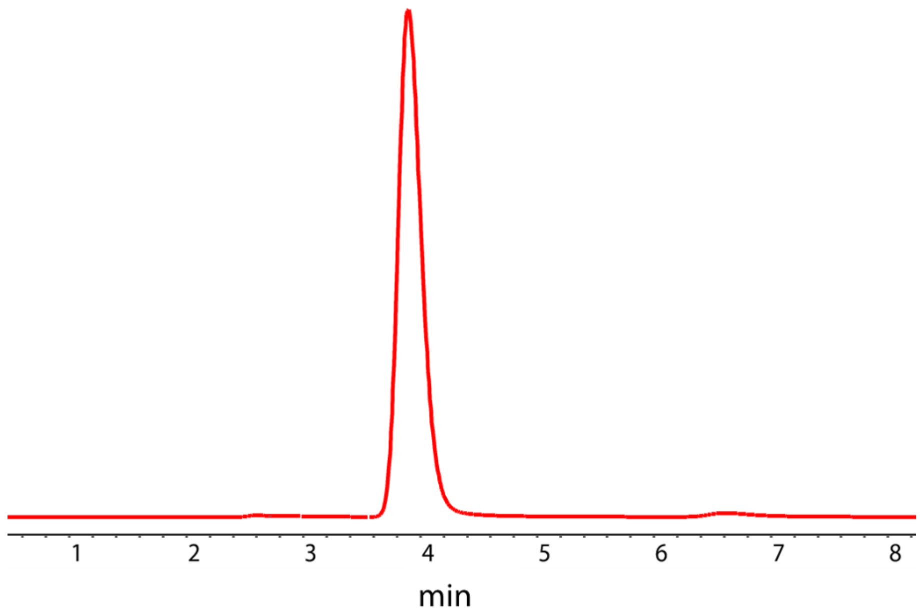

Figure S31. Analytical HPLC trace at 329 nm absorbance wavelength of HINA using H_2O as the eluent.

9. NMR measurement

9.1. 3-hydroxy-isonicotinic aldehyde (HINA)

9.1.1. DMSO-d_6

Table S9. ^{1}H chemical shifts of HINA in DMSO-d_6.

	a	b	c	d	e
^{1}H/ppm	8.19, d	7.46, d	10.37,s	11.06, s (-OH)	8.46, s

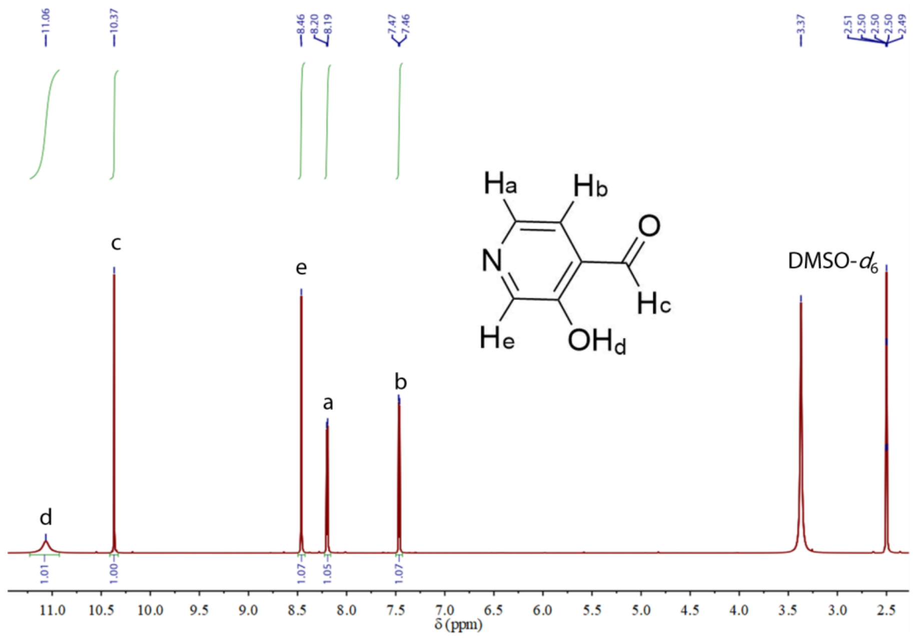

Figure S32. ^{1}H NMR spectrum of HINA in DMSO-d_6.

Table S10. ^{13}C chemical shift of HINA in DMSO-d_6.

	1	2	3	4	5	6
^{13}C/ppm	140.53	119.66	126.68	154.55	141.56	190.37

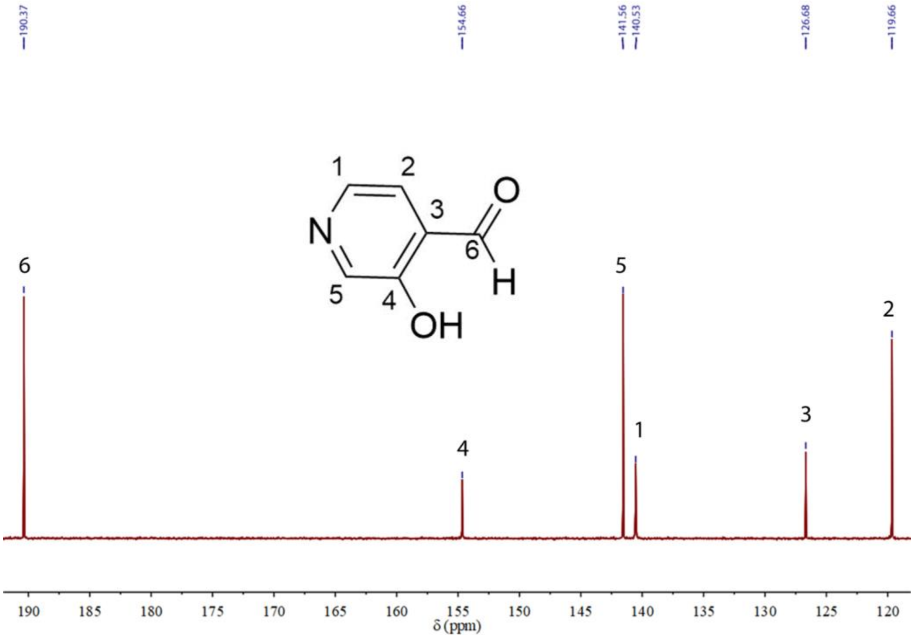

Figure S33. ^{13}C NMR spectrum of HINA in DMSO-d_6.

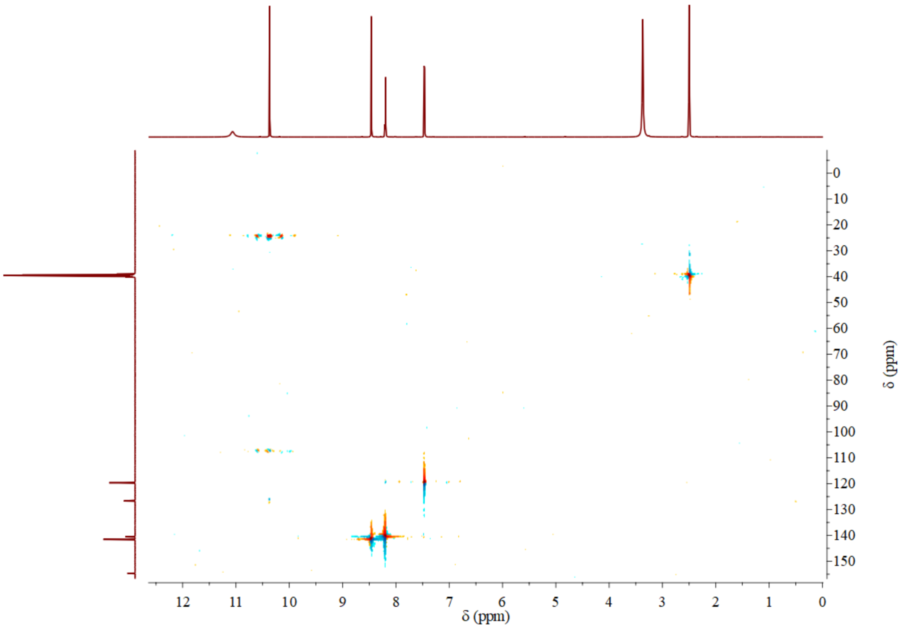

Figure S34. HSQC spectrum of HINA in DMSO-d_6.

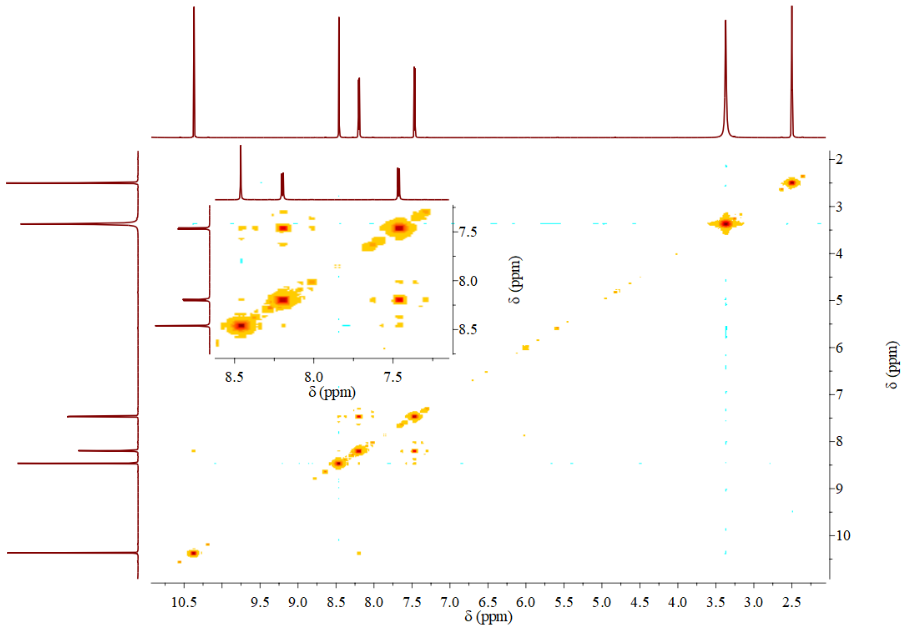

Figure S35. COSY spectrum of HINA in DMSO-d_6.

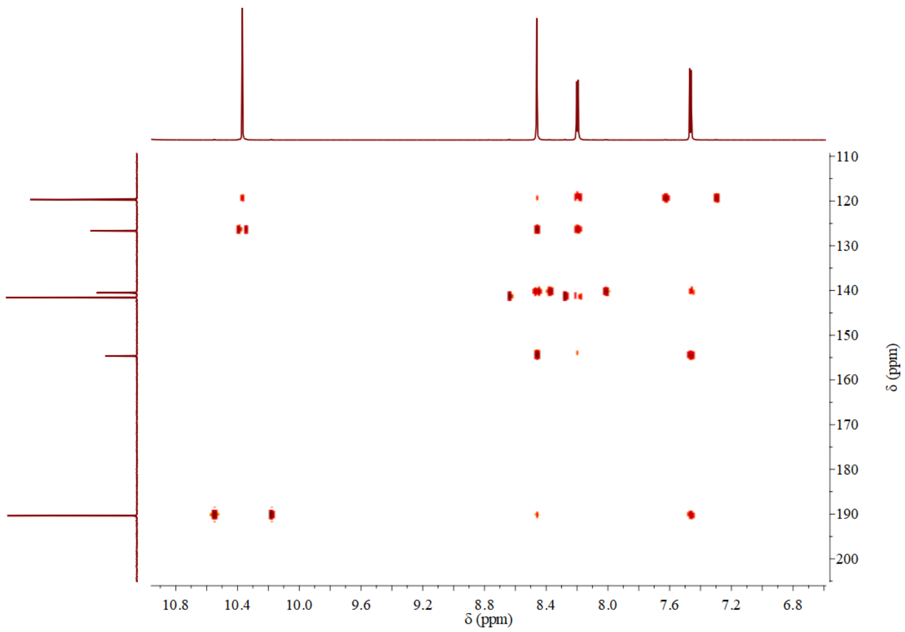

Figure S36. HMBC spectrum of HINA in DMSO-d_6.

9.1.2. NMR in a solvent mixture of DMSO-d_6 with D_2O

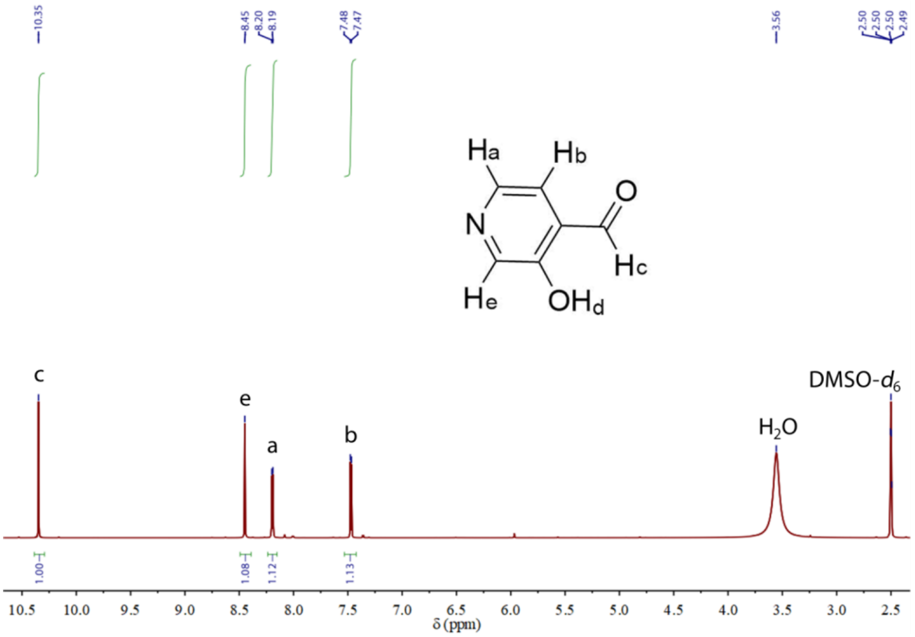

Figure S37. ^1H NMR spectrum of HINA in a mixture solvent of DMSO-d_6 and D_2O (v/v, 24/1).

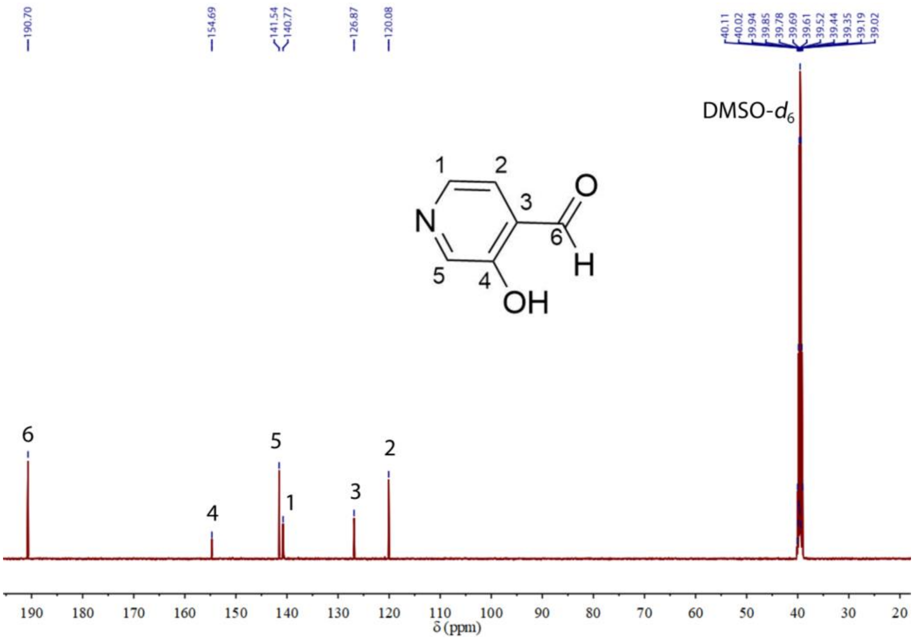

Figure S38. ^{13}C NMR spectrum of HINA in a mixture solvent of DMSO-d_6 and D_2O (v/v, 24/1).

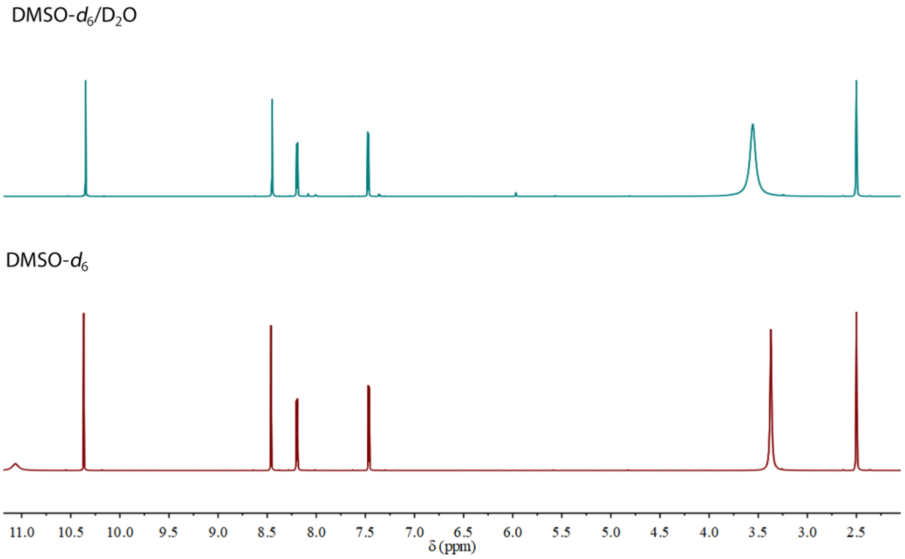

Figure S39. ^{1}H NMR comparation of HINA in DMSO-d_6 (bottom, red line) and the mixture solvent of DMSO-d_6 and D_2O (top, blue line).

9.1.3. Acetonitrile-d_3 (ACN-d_3)

Table S11. [1]H chemical shift of HINA in ACN-d_3.

	a	b	c	d	e
^{1}H/ppm	8.34, d	7.58, d	10.08, s	10.06, s (-OH)	8.46, s

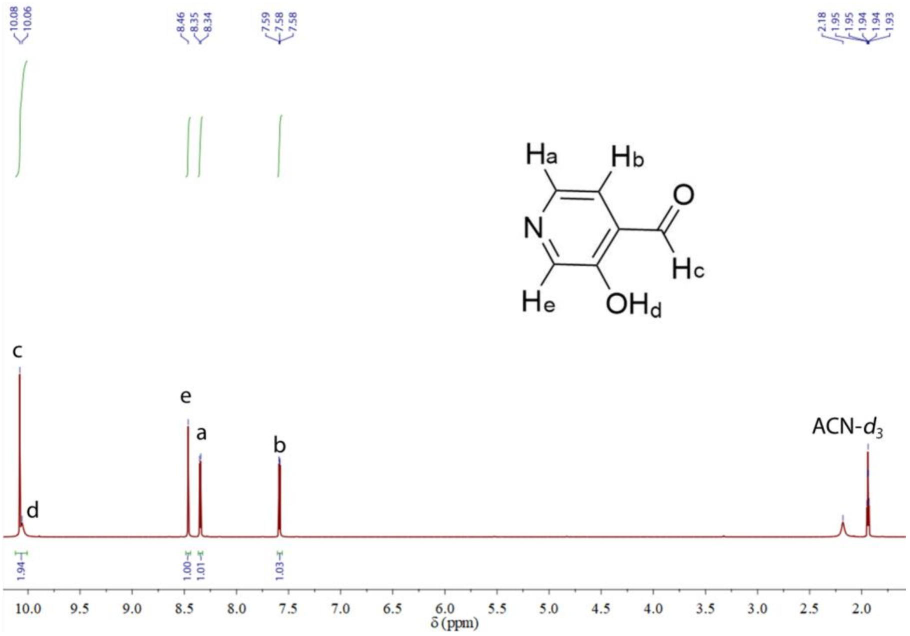

Figure S40. 1H NMR spectra of HINA in ACN-d3.

Table S12. ^{13}C chemical shift of HINA in ACN-d_3.

	1	2	3	4	5	6
^{13}C/ppm	142.30	124.97	125.46	155.10	142.40	198.19

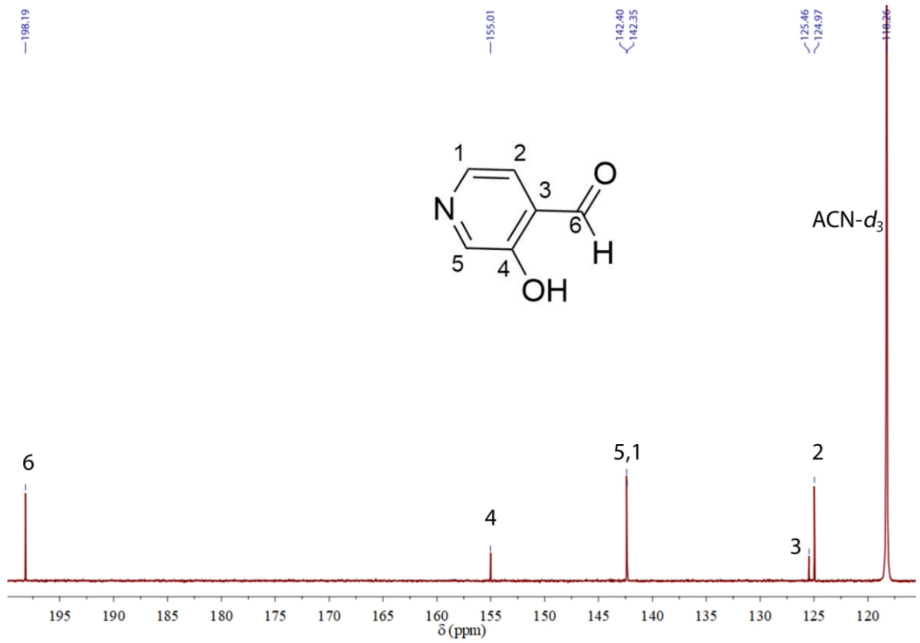

Figure S41. ^{13}C NMR spectra of HINA in ACN-d_3.

9.1.4. Deuterium oxide (D_2O)

Hydrate formation constant (K_{hyd}) calculation was carried out via:[9-12]

$$K_{hyd} = \frac{\text{peak area integration of HINA} - \text{hydrate}}{\text{peak area integration of HINA}}$$

The more electron-poor neutral HINA has a higher tendency to form hydrates ($K_{hyd} = 8{\cdot}10^{-3}$ M^{-1} for R-CHO + H_2O ↔ R-CH(OH)$_2$ from NMR integration) than its anion ($2{\cdot}10^{-3}$ M^{-1}).

Table S13. The proton chemical shift of HINA and HINA-hydrate in D_2O.

	a (a’)	b (b’)	c (c’)	d (d’)	e (e’)
HINA	7.91-7.93	7.70-7.71, d	10.19, s	-	8.18, s
HINA-hydrate	7.91-7.93	7.61-7.62, d	6.22, s	-	7.93

Table S14. The carbon chemical shift of HINA-hydrate in D_2O.

	1 (1’)	2 (2’)	3 (3’)	4 (4’)	5 (5’)	6 (6’)
HINA	133.65	124.55	160.78	130.20	140.19	194.13
HINA-hydrate	133.65	122.17	157.21	141.98	133.65	86.05

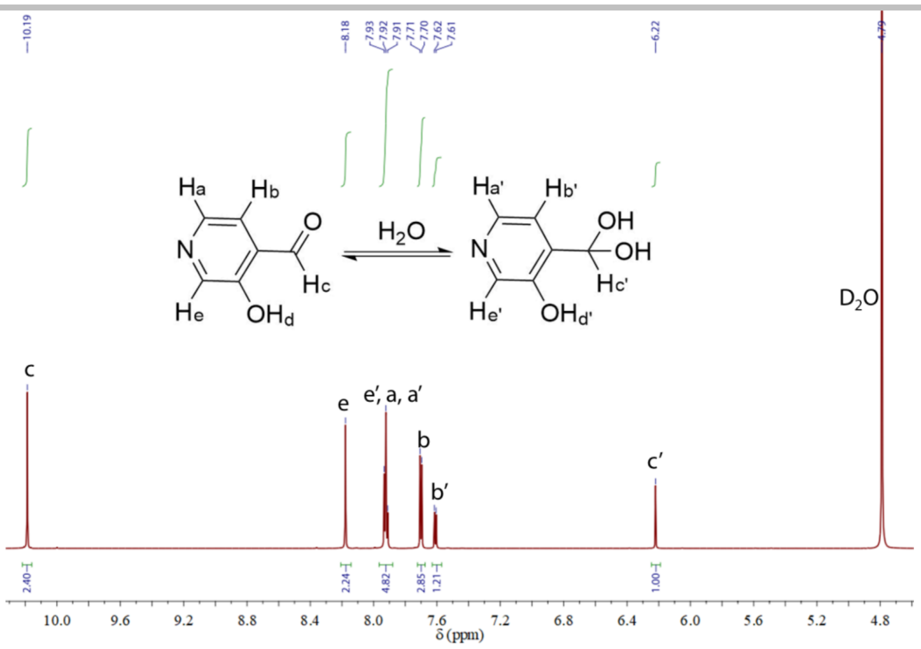

Figure S42. ^{1}H NMR spectra of HINA in D_2O.

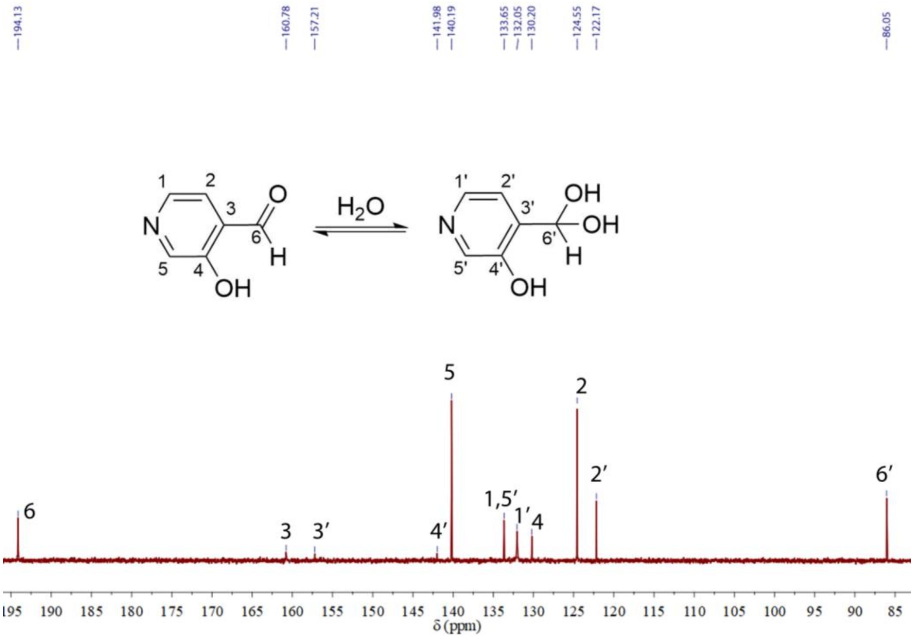

Figure S43. ^{13}C NMR spectra of HINA in D_2O.

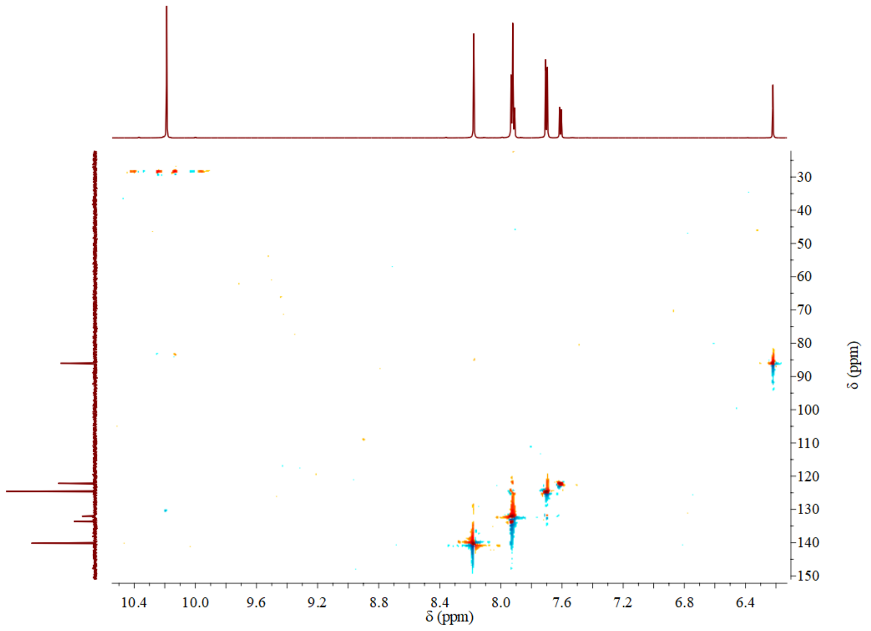

Figure S44. HSQC spectra of HINA in D_2O.

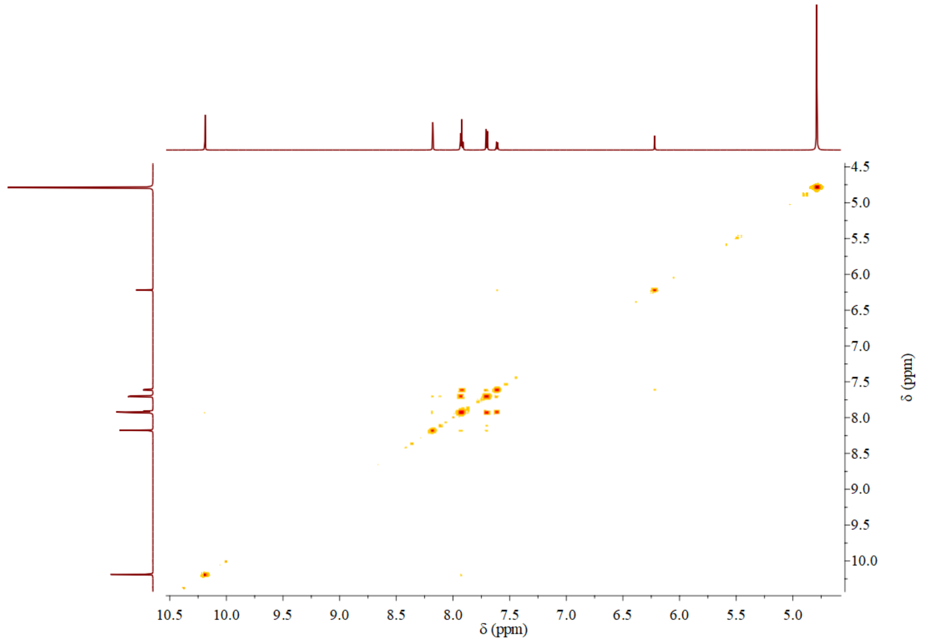

Figure S45. COSY spectra of HINA in D_2O.

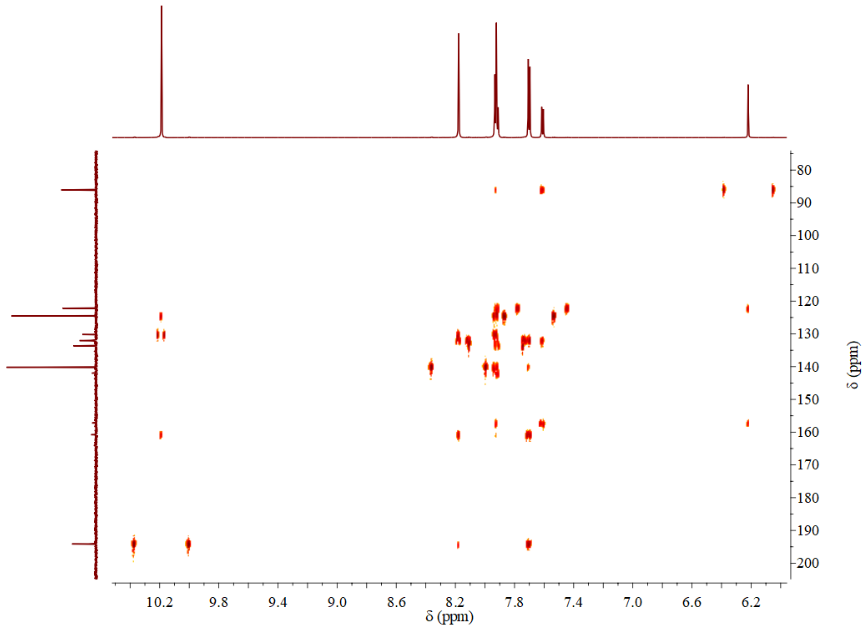

Figure S46. HMBC spectra of HINA in D_2O.

9.1.5. Deuterium oxide (D_2O) under basic conditions

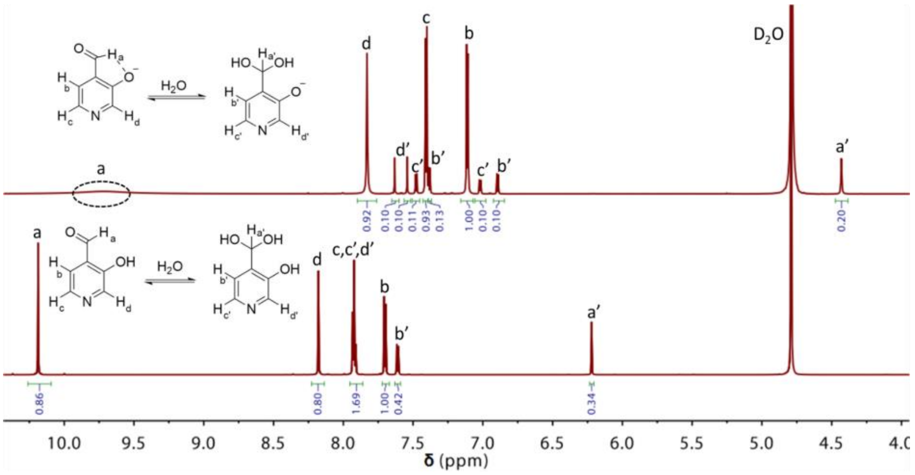

Figure S47. ^{1}H NMR spectra of HINA in D_2O (bottom) and with NaOH addition (top).

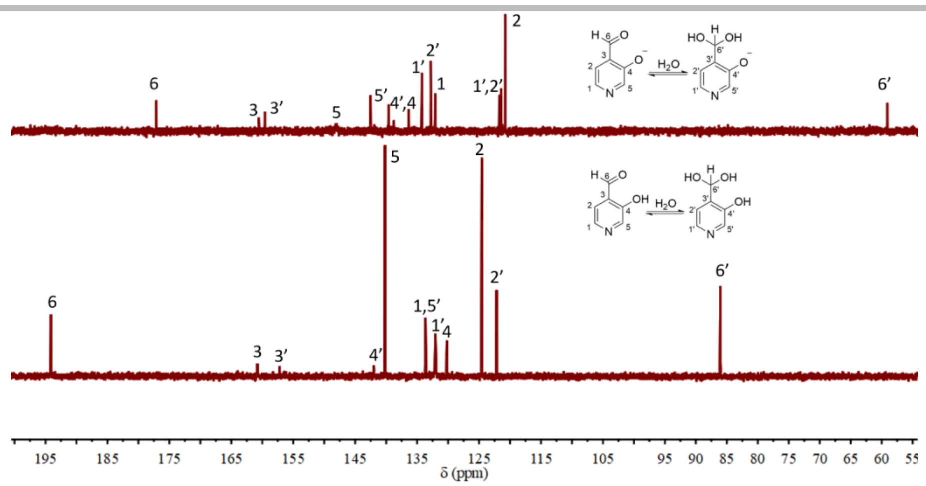

Figure S48. ^{13}C NMR spectra of HINA in D_2O (bottom) and with NaOH addition (top).

9.1.6. hexafluoro-2-propano (HFP)

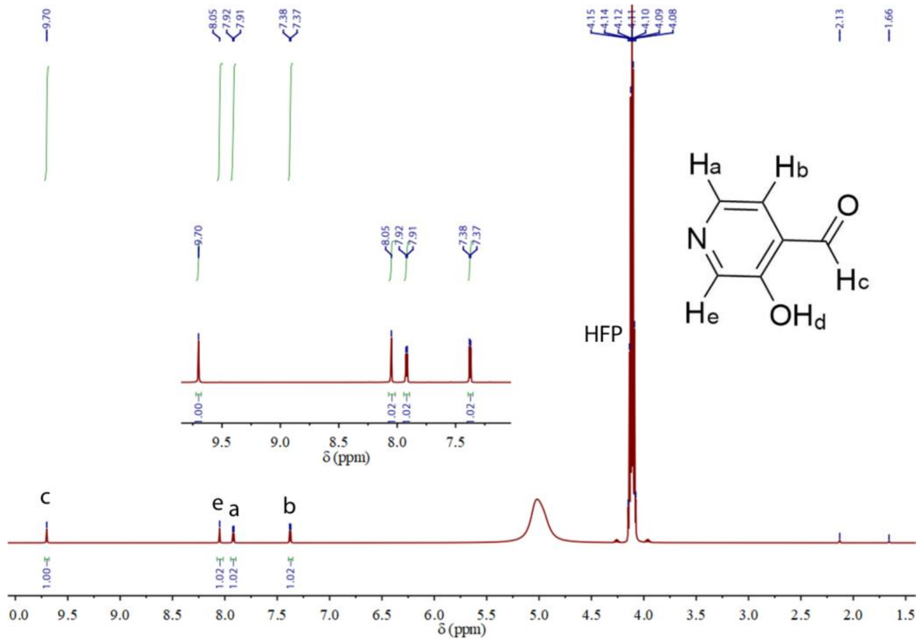

Figure S49. ^{1}H NMR spectra of HINA in a solvent mixture of HFP and ACN-d_3 (v/v = 24/1). The insert graph is the zoom of the spectra in the downfield.

9.2. 3-hydroxy-picolin aldehyde (HPA)

9.2.1. ACN-d_3

Table S15. ^{1}H chemical shift of HPA in ACN-d_3.

	a	b	c	d	e
^{1}H/ppm	8.35-8.36	7.52-7.54	7.40-7.42	10.01	10.59

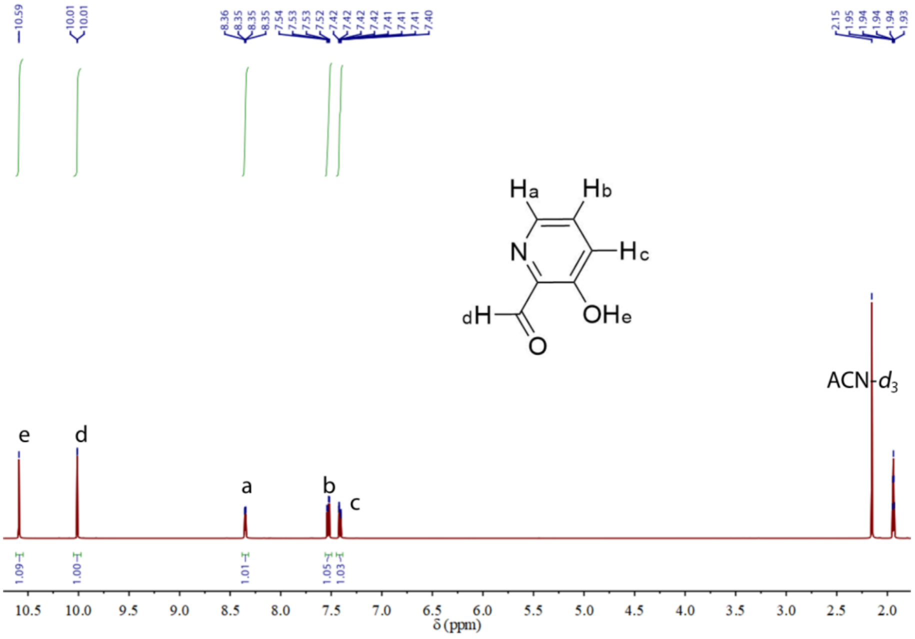

Figure S50. ^{1}H NMR spectrum of HPA in ACN-d_3.

Table S16. ^{13}C chemical shift of HPA in ACN-d_3

	1	2	3	4	5	6
^{13}C/ppm	137.92	131.31	126.67	159.12	143.61	200.09

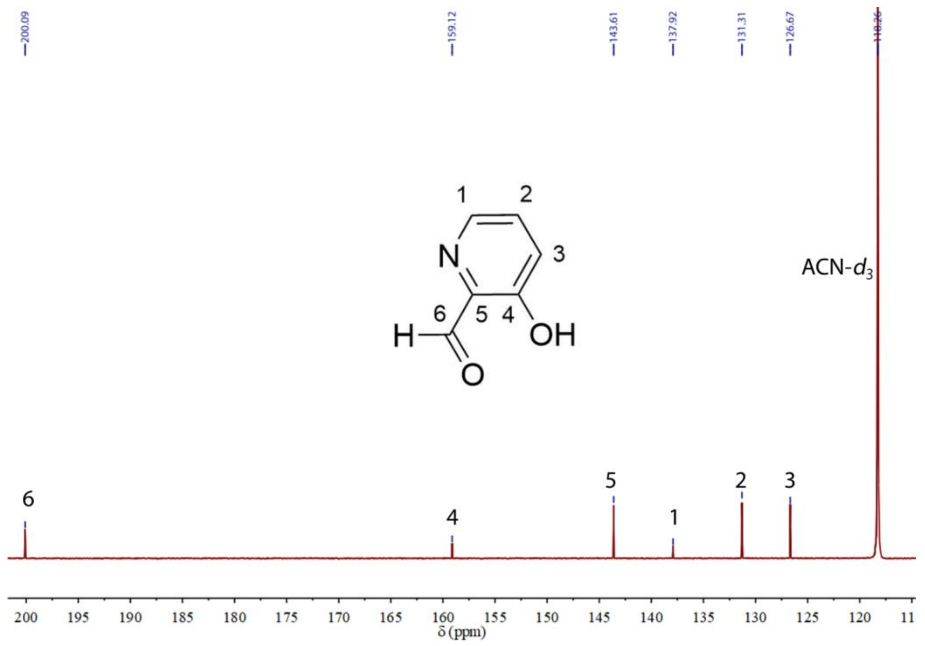

Figure S51. ^{13}C NMR spectrum of HPA in ACN-d_3.

9.2.2. Deuterium oxide (D_2O)

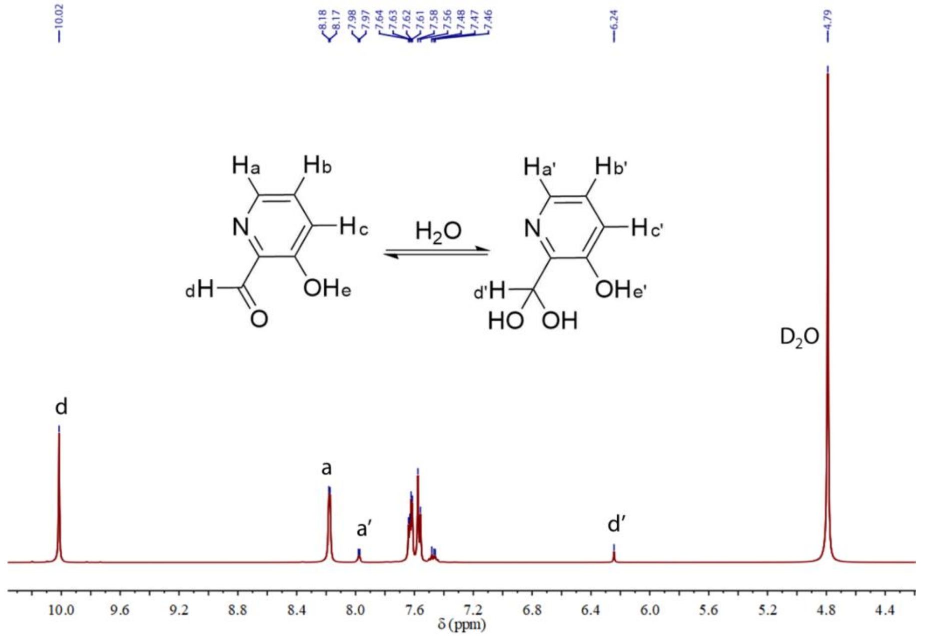

Figure S52. ^{1}H NMR spectra of HPA in D_2O, the highlight peak with black color is the peaks from HPA-hydrate.

9.3. 3-hydroxy-4-acetyl pyridine (HAP)

9.3.1. ACN-d_6

Table S17. ^{1}H chemical shift of HAP in ACN-d_3.

	a	b	c	d	e
^{1}H/ppm	8.21-8.22, d	7.63-7.64, d	2.62, s	11.44, s	8.39, s

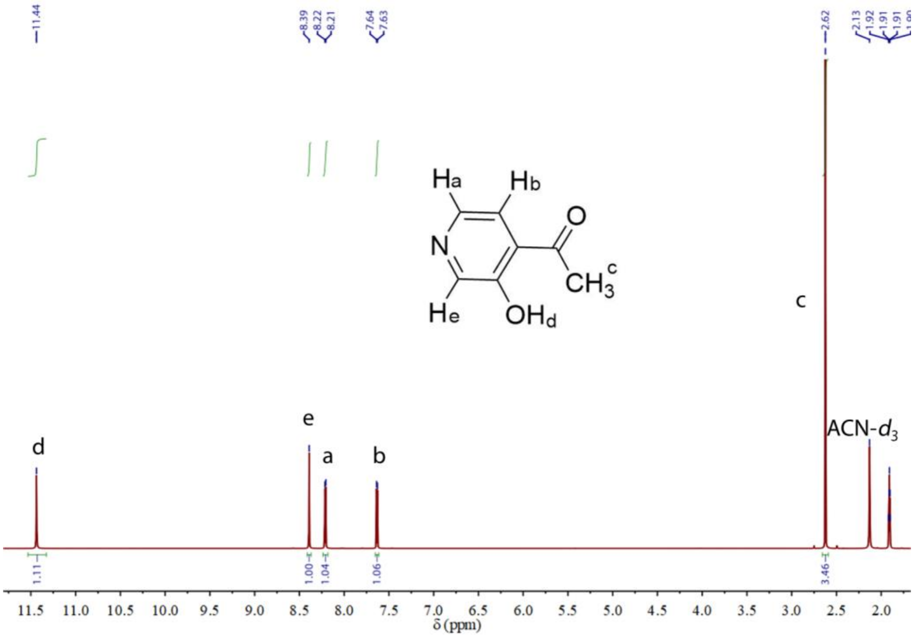

Figure S53. ^{1}H NMR spectrum of HAP in ACN-d_3.

Table S18. ^{13}C chemical shift of HAP in ACN-d_3

	1	2	3	4	5	6	7
^{13}C/ppm	143.23	123.42	124.57	155.94	141.44	207.07	27.37

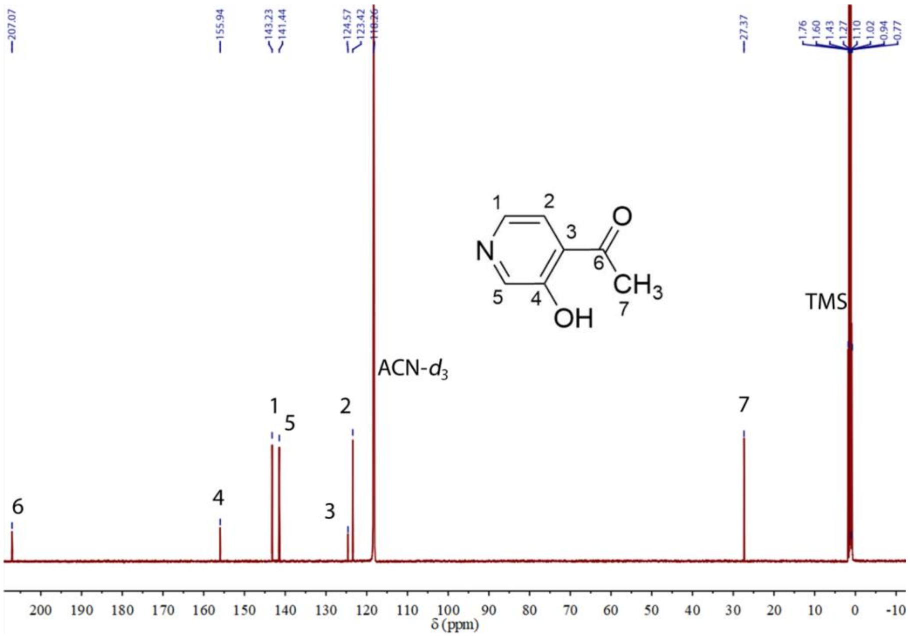

Figure S54. ^{13}C NMR spectrum of HAP in ACN-d_3.

9.3.2. Deuterium oxide (D_2O)

Table S19. ^{1}H chemical shift of HAP in D_2O.

	1	2	3	4	5	6	7
^{1}H/ppm	8.10-8.11, d	7.73-7.74, d	-	-	8.27, s	-	2.66-2.68, m

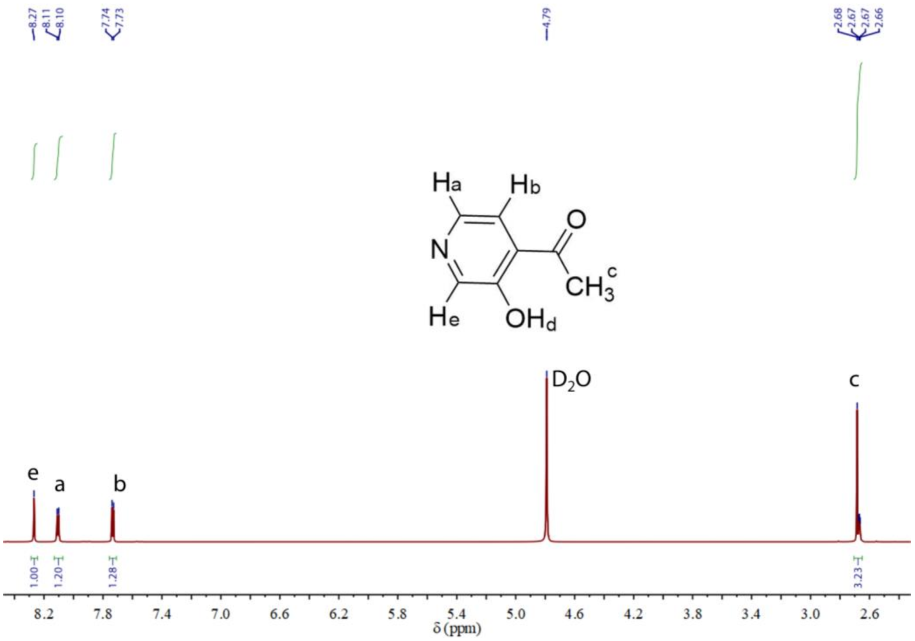

Figure S55. ^{1}H NMR spectra of HAP in D_2O.

10. HINA indicator displacement assay with Platinum (Pt) complex

10.1. Assay in organic solvent

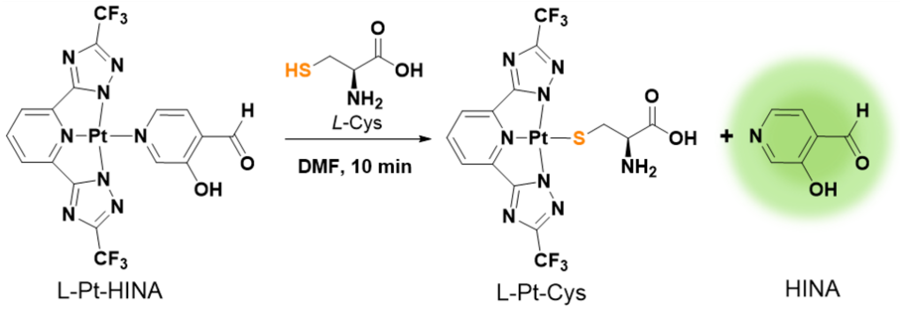

Scheme S1. Schematic of representation of HINA can be released as an emissive indicator upon addition of *L*-Cys in the solution of L-Pt-HINA complex in DMF at 25 °C

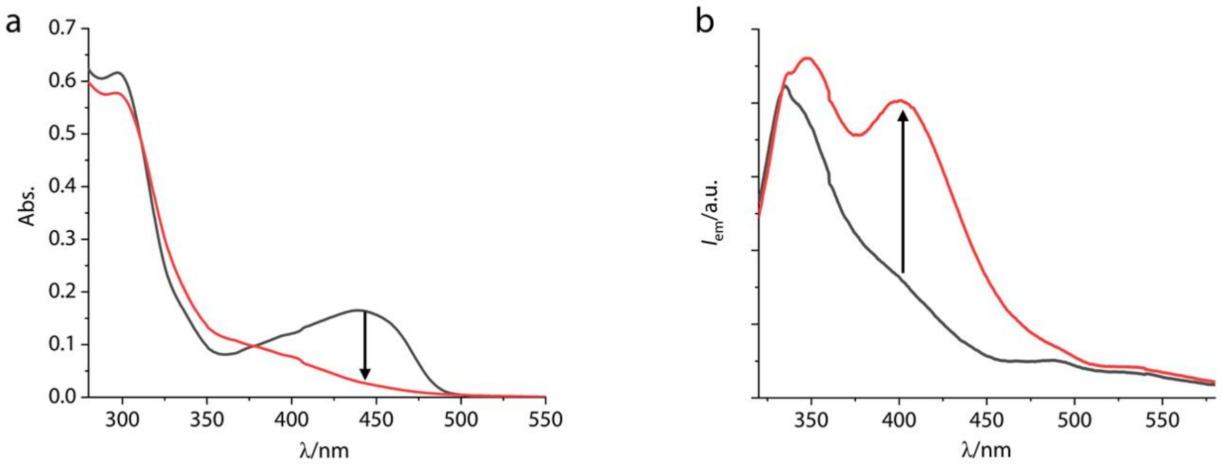

Figure S56. Absorbance (a) and emission (b) spectra of *L*-Cys (50 μM) upon addition L-Pt-HINA (50 μM, balck line) in DMF at 25 °C

10.2. Assay in aqueous media

10.2.1. MS spectra

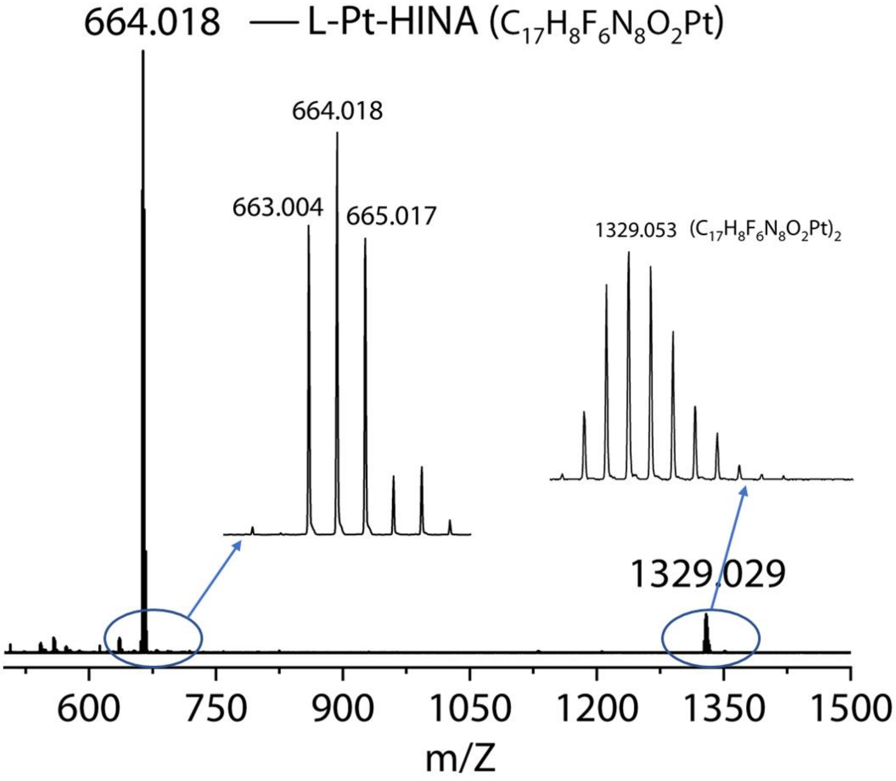

Figure S57. ESI-MS spectra of the solution of HINA (50 μM) after reacting with L-Pt-DMSO (55 μM) in 25 mM $NaHCO_3$ buffer (pH 9.5) contanining 0.9 mM CTAB. Calcd. for $[M]^-$: 664.030; $[2M-H]^-$: 1329.060; found: $[M]^-$: 664.018; $[2M-H]^-$: 1329.029.

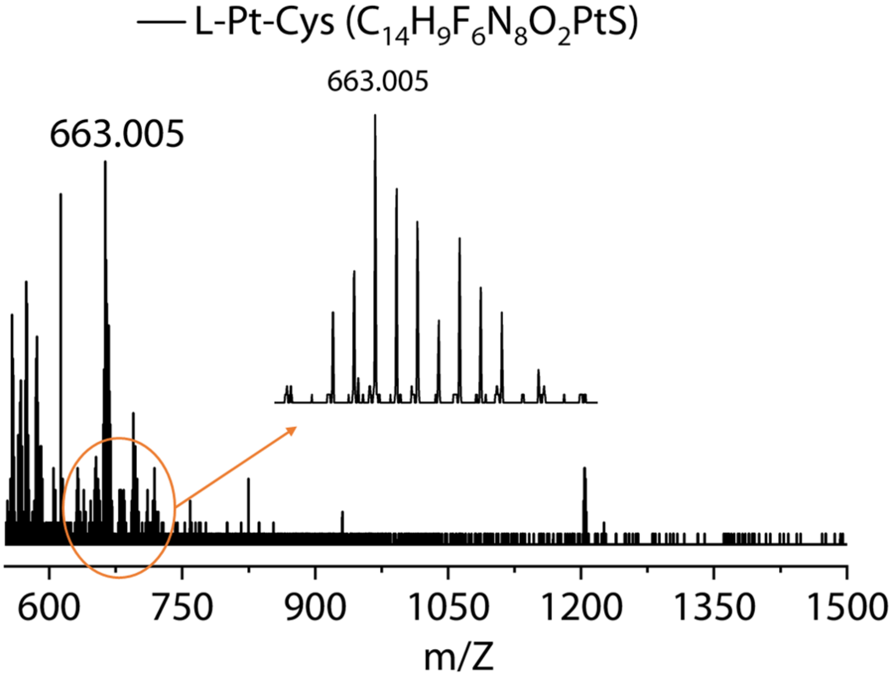

Figure S58. ESI-MS spectra of the solution of *L*-Cys (50 μM) after reacting with L-Pt-HINA (50 μM) in 25 mM $NaHCO_3$ buffer (pH 9.5) contanining 0.9 mM CTAB. Calcd. for $[M]^+$: 663.012; found: $[M]^+$: 663.005.

10.2.2. Binding constant determination

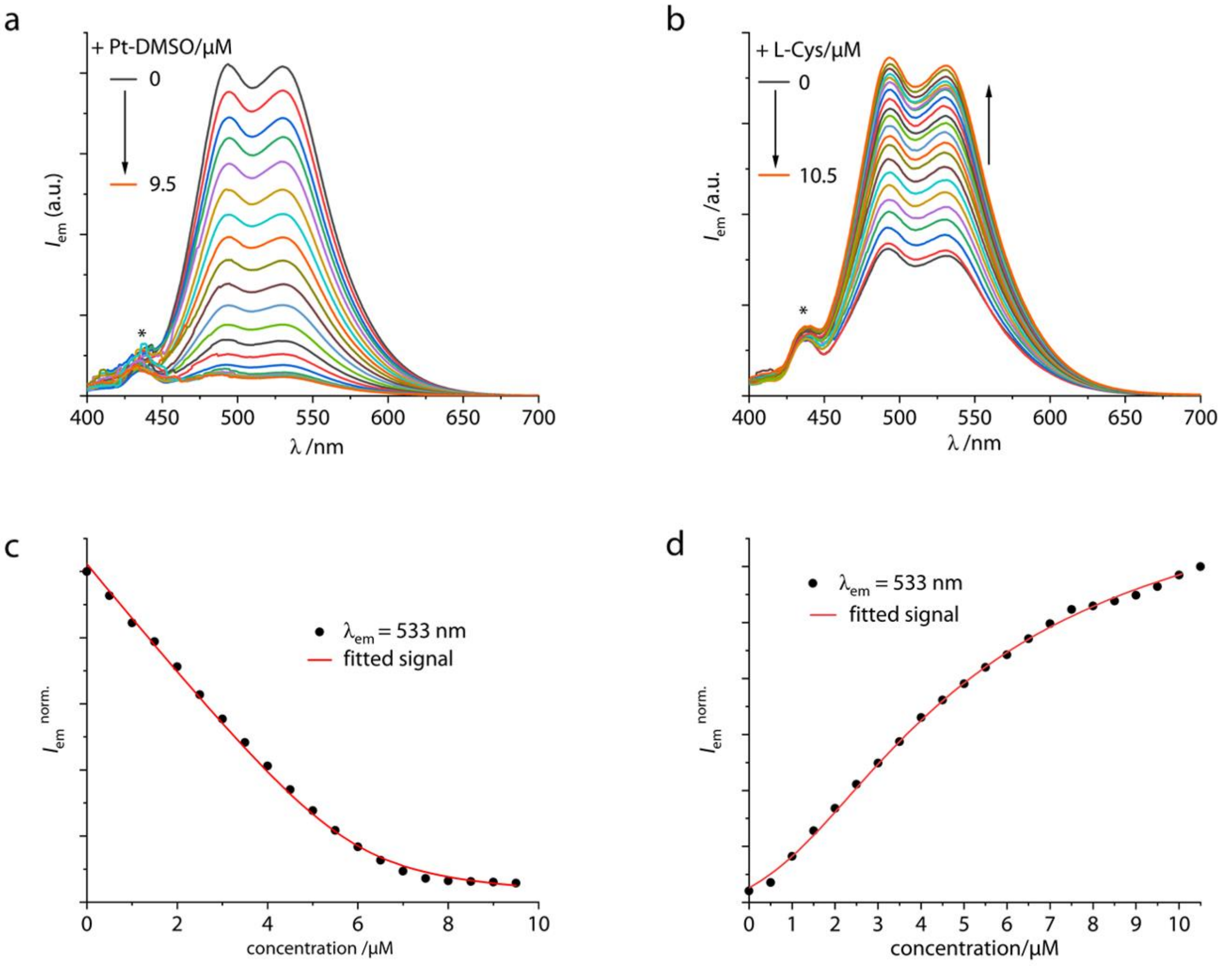

Figure S59. a) Emission spectra of HINA (5 μM, λ_{ex} = 380 nm) was titrated by L-Pt-DMSO with a concentration step of 0.5 μM. b) Emission spectra of L-Pt-HINA (5 μM, λ_{ex} = 380 nm) was titrated by *L*-Cys with a concentration step of 0.5 μM. c) Binding constant determined by fluorescence intensity (λ_{ex} = 380 nm, λ_{em} = 533 nm) of HINA (5 μM) and L-Pt-DMSO (0-9.5 μM). The binding constant of HINA to L-Pt-DMSO is K_{a1} = 5.0 (± 0.2) 10^6 M^{-1} with a direct binding model, d) Binding constant determined by fluorescence intensity (λ_{ex} = 380 nm, λ_{em} = 533 nm) of L-Pt-HINA (5 μM) and *L*-Cys (0-10.5 μM). The binding constant of *L*-Cys with Pt-DMSO is K_{a2} = 1.0 (± 0.2) 10^7 M^{-1} by using an indicator displacement assay fitting model, the K_{a1} value was taken from the first step as a K_{HD} value in the fitting process . Acquired data is depicted as black dots and fitted data as red line.

10.3. NMR spectra

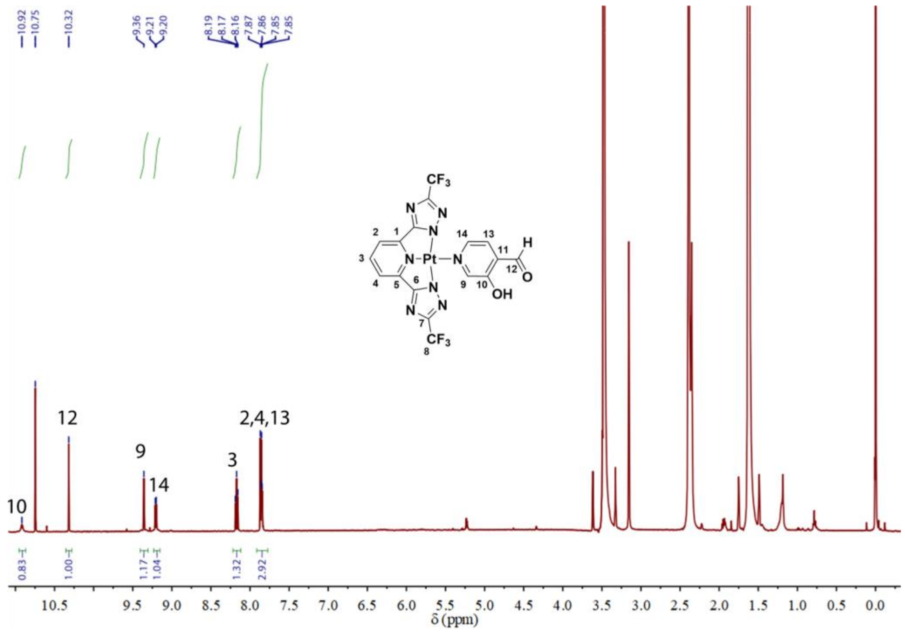

Figure S60. The ^{1}H NMR spectrum of L-Pt-HINA in THF-d_8. The peaks are not assigned in the graph are coming from the solvent of THF-d_8. The ^{1}H NMR of THF-d_8 alone is shown as a reference in Figure S60.

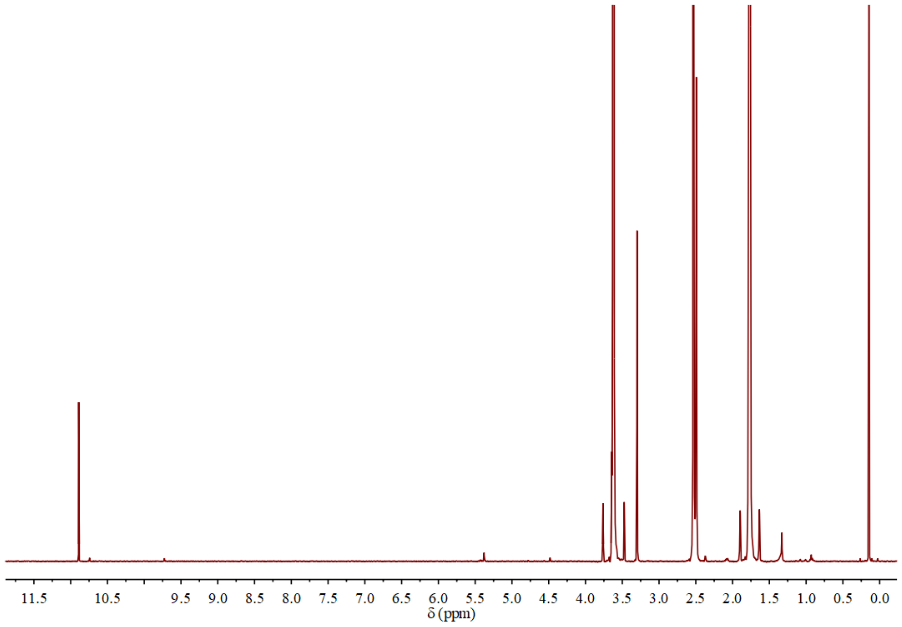

Figure S61. ^{1}H NMR spectrum of THF-d_8.

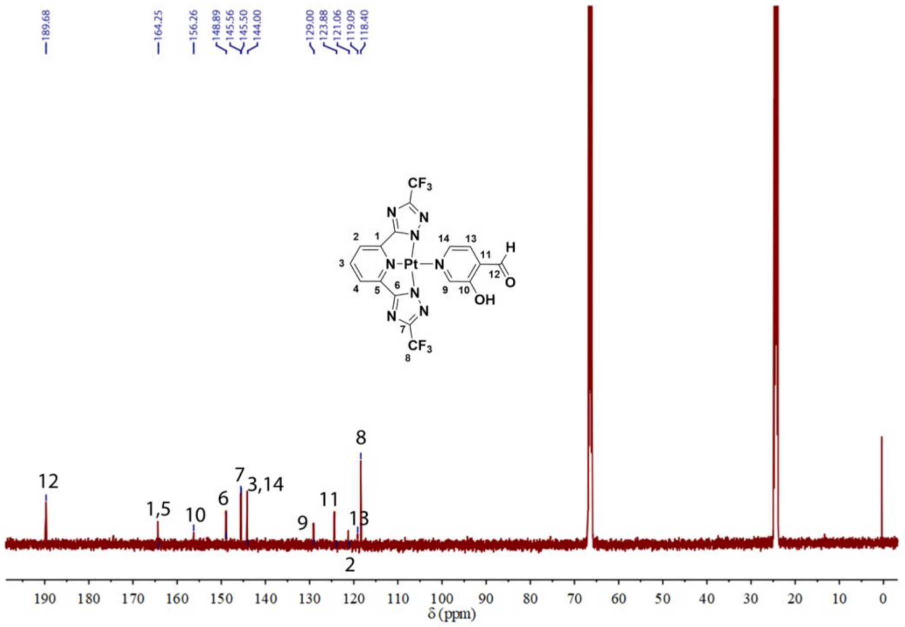

Figure S62. The ^{13}C NMR spectrum of probe in THF-d_8.

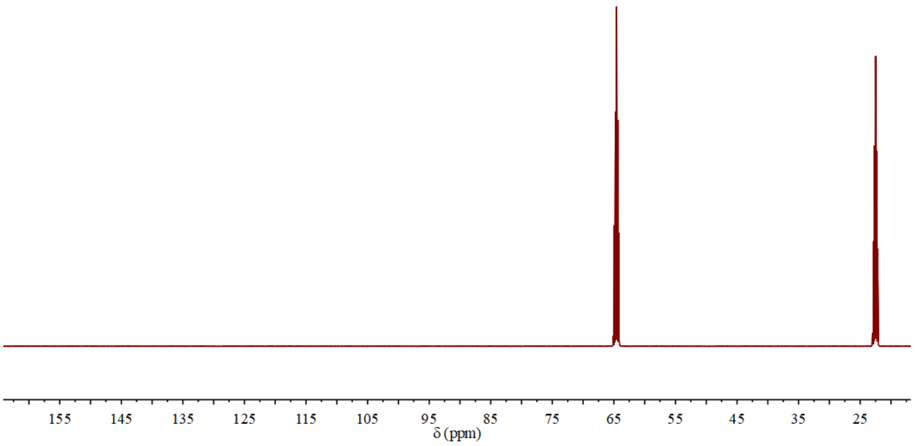

Figure S63. ^{13}C NMR spectrum of THF-d_8.

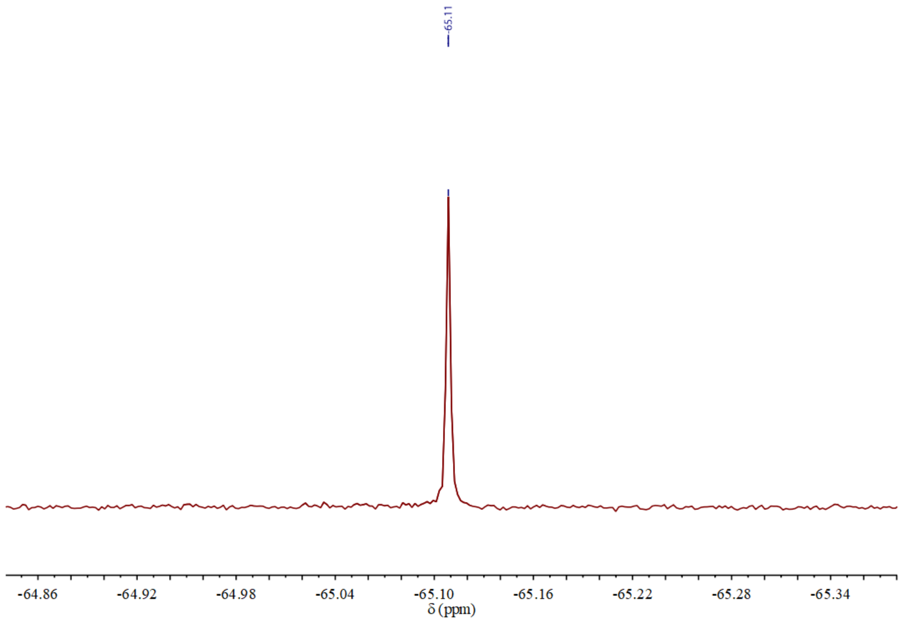

Figure S64. The ^{19}F NMR spectrum of the probe in THF-d_8.

11. HINA indicator displacement assay with palladium (Pd) complex

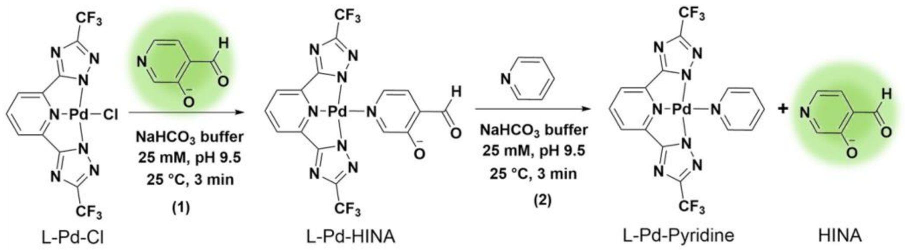

Scheme S2. Schematic of representation of HINA can be reacted with Pd(II) precursor to form a non-emissive L-Pd-HINA complex, and HINA can be released as an emissive indicator upon addition of pyridine in $NaHCO_3$ buffer (25 mM) with 0.9 mM CTAB at pH 9.5 and 25 °C

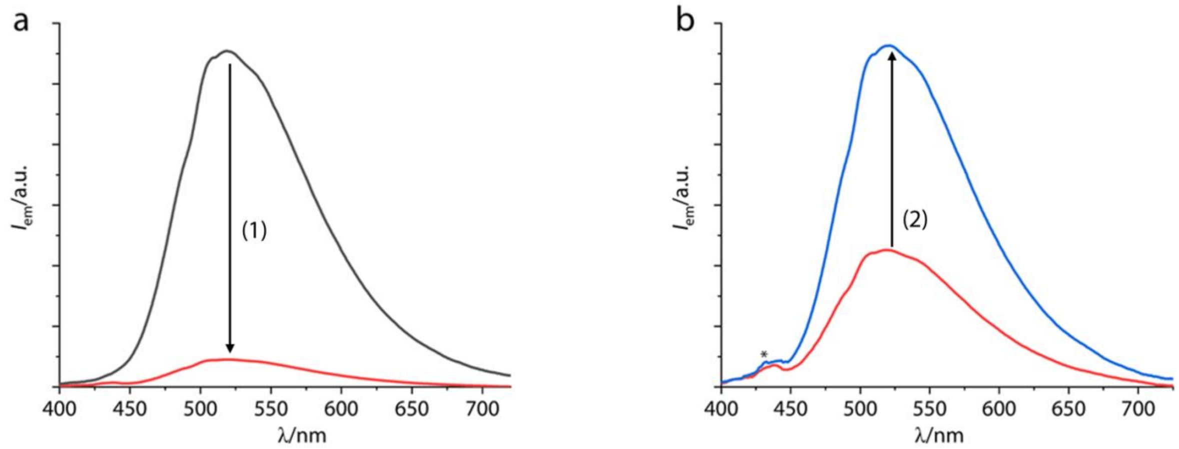

Figure S65. Emission spectra of the formation of non-emissive L-Pd-HINA complex (a) and upon addition of pyridine in $NaHCO_3$ buffer (25 mM) with 0.9 mM CTAB at pH 9.5 and 25 °C. The asterisk in the emission spectrum indicates the Raman scattering band of water at the selected excitation wavelength.

12. Cysteine sensing with HINA in aquous media

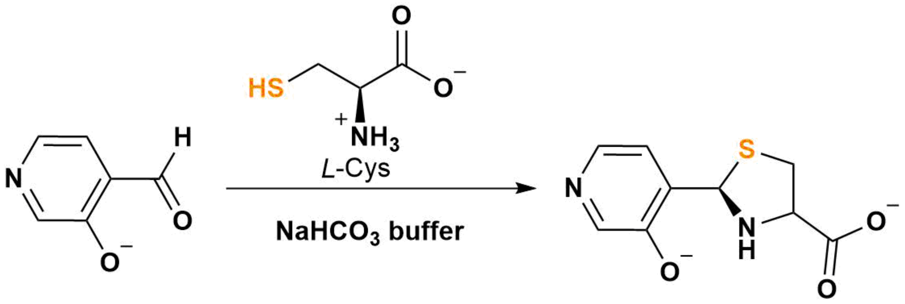

Scheme S3. Schematic of representation of the reaction of L-Cysteine with HINA in $NaHCO_3$ buffer (25 mM) at pH 9.5 and 25 °C.

12.1. spectra measurement

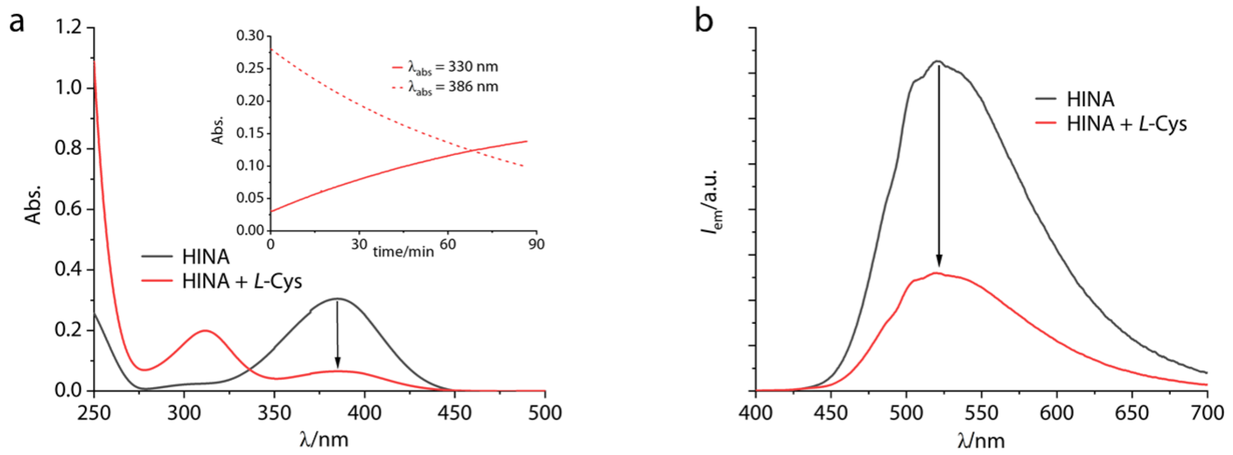

Figure S66. Absorbance (a) and emission (b) spectra of HINA (50 µM, black line) upon addition of L-cysteine (1 mM, red line) in NaHCO3 buffer (25 mM) at pH 9.5 and 25 °C. Inset shows the absorbance kinetics trace of the reaction. The absorbance and emission spectra were recorded after 90 minutes of reaction time.

12.2. NMR spectra

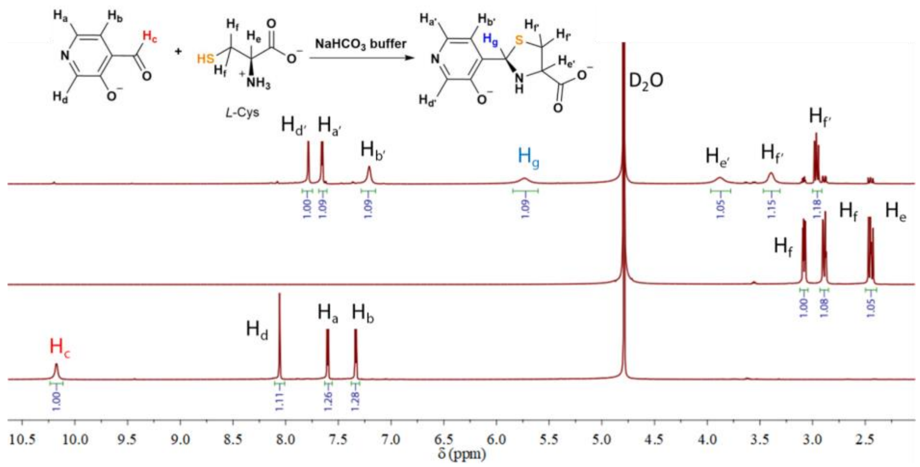

Figure S67.^{1}H NMR spectra of HINA (50 mM, bottom), *L*-Cys (50 mM, middle) and the mixture solution of HINA and *L*-Cys after overnight reaction (top) in D_2O under basic condition. Peak assignements were carried out with the help of 2D NMR spectra.

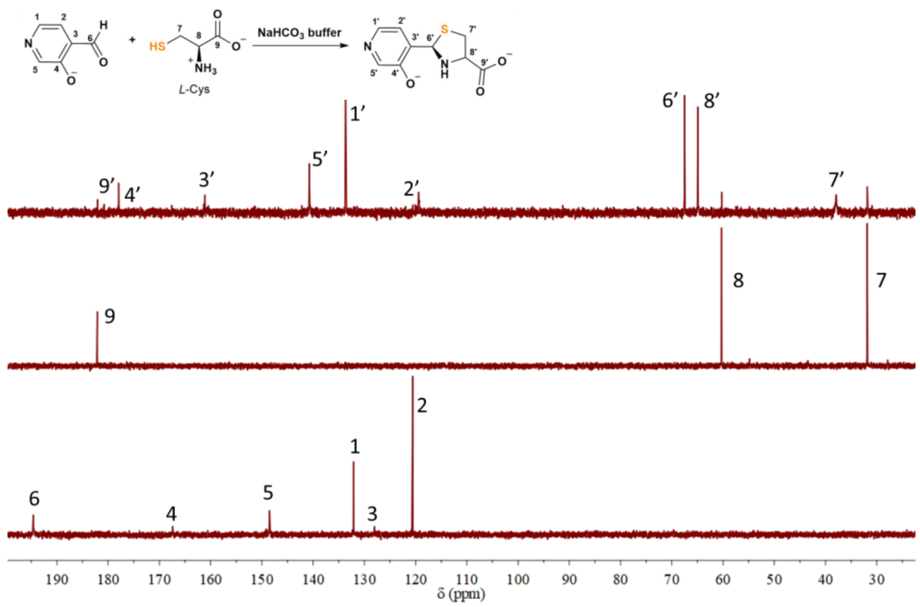

Figure S68. ^{13}C NMR spectra of HINA (50 mM, bottom), *L*-Cys (50 mM, middle) and the mixture solution of HINA and *L*-Cys after overnight reaction (top) in D_2O under basic condition. Peak assignements were carried out with the help of 2D NMR spectra.

13. Cell experiment

Cells were grown as a single layer in adhesion in an appropriate cell culture medium and were maintained at 37 °C and in a 5% CO_2 humidified atmosphere.

MC38 cells were grown in DMEM-Low Glucose (Dulbecco′s Modified Eagle Medium, pyruvate and L-glutamine, Gibco) with the addition of 10% fetal bovine serum (FBS, Gibco) 0.1 mM MEM Non-essential Amino Acid Solution (Sigma-Aldrich) 10 mM HEPES buffer (Sigma-Aldrich) 100 U/mL penicillin 0.1 mg/mL streptomycin. MCF10 cells were grown in DMEM-Low Glucose (Dulbecco's Modified Eagle Medium, pyruvate and L-glutamine, Gibco) with the addition of 10% of fetal bovine serum (FBS) and 1% L-glutamine (200 mM) 100 U/mL penicillin, 0.1 mg/mL streptomycin, 100 ng/mL cholera toxin (Sigma-Aldrich) and 10 µg/mL human insulin (Sigma-Aldrich). HEK293 cells were grown in DMEM-Low Glucose (Dulbecco′s Modified Eagle Medium, pyruvate and L-glutamine, Gibco) with the addition of 10% fetal bovine serum (FBS) 100 U/mL penicillin, 0.1 mg/mL streptomycin.

MC38, MCF10, and HEK293 were seeded on round glass slide in 12-well plates at the concentration of 70000 cell/well and left to adhere for 24 hours. Exclusively for HEK293 cells, round glass slide was pre-coated with 0.01% poly-lysine solution (Santa Cruz) to optimize the cell attachment.

HINA, HPA and HAP internalization was carried out incubating at the concentration of 100 µM for 1 hour. After incubation, cells were washed two times with Phosphate-Buffered Saline (PBS 1x, Gibco) fixed with 4% paraformaldehyde (PAF) in PBS 1x for 20 minutes and washed two times with PBS 1x. Images were captured with LSM 710 confocal microscopy (Zeiss) equipped with specific laser λ_{ex} = 405 nm. The images of HEK 298 and MCF cell line were acquired in a 60% power of laser, gain 873, digital gain 0.89 and offset -285; the image of MC38 cell line were collected in a 18 % power of lase, gain 716, digital gain 1.43 and offset -257.

In order to evaluate the cytotoxicity of pH molecules, cell viability assay (CellTiter96® Aqueous One solution Cell Proliferation Assay, Promega) was carried out on MCF10 and HEK293 cell lines. Cells were seeded at 20000 cells/well into 96-well plate. The fresh medium containing serial concentrations of HINA or HPA or HAP (10 µM - 200 µM) was added 24 hours after the seeding. Cells were incubated with the treatment for 24 hours. CellTiter96® Aqueous reagent (20 µL/well) was added and incubated for 2 hours according to manufacturer's procedure. At least, plates were placed in a microplate reader (Multiskan GO, Thermo Scientific) to measure the absorbance at 490 nm, and the cytotoxicity was expressed as % of cell viability compared to control.

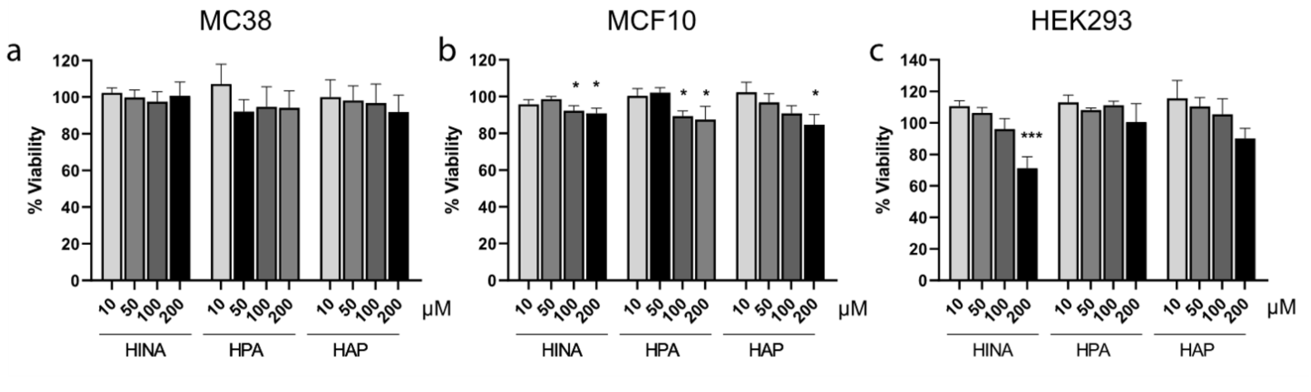

Figure S69. Cytotoxicity assay of HINA, HPA, and HAP in a) MC38, b) MCF10 and c) HEK293 cell line after 24 hours of incubation at increasing concertations (10 -200 µM). The cytotoxicity are expressed as % of cell viability compared to control. Data are expressed as mean ± standard error. One-way ANOVA and Dunnett's post hoc test *p< 0.05 and ***p<0.0005.

Statistical analysis

Statistical analysis was done using Prism Software (Prism 8.0; GraphPad Software, La Jolla, CA). One-way ANOVA followed Dunnett's post hoc test was applied for data analyses. For all analyses a p value <0.05 was considered statistically significant.

14. Photoactivation experiments

14.1. Photoactivation in cell

U2OS or HeLa cells plated on glass coverslips were incubated with 2 mM HINA 30 – 60 minutes in DMEM without phenol red (Gibco) supplemented with Pen/Strep (AppliChem) at 37°C, 5% CO_2 and 95% humidity. Washing (when applicable) and imaging were performed in the same medium. Imaging was performed at room temperature on an Abberior easy3D STED/RESOLFT QUAD scanning microscope (Abberior Instruments) built on a motorized inverted microscopeIX83 (Olympus) and equipped with a 100x/1.40 UPlanSApo oil immersion objective lens (Olympus). For confocal experiments, cells were imaged and photoactivated with a 405 CW laser set to 3% of power, which corresponds to ~100uW before the back focal aperture, and detection was performed with avalanche photodiodes (APD) in spectral window 475-600 nm. For the photoactivation, selected regions were scanned 100 times with 250 nm pixel size, 10 µs dwell time, and 1 line accumulation.

Quantification of the fluorescence intensity before and after photoactivation was performed in ImageJ 1.50b. For each irradiated cell, 5 different regions of interest (ROIs, 3 x 3 µm) in which no obvious cellular structures were visible (e.g. no mitochondria-like labeling) were selected and mean intensity values were averaged. As control, 5 ROIs from the same field of view and in non-irradiated cells were similarly selected and averaged. All irradiated cells (5/5) showed an increase in fluorescence in no-wash conditions, while 3/5 cells glowed up in case of washing.

Widefield imaging was performed on the same microscope using a Basler acA4112-20 µm camera, X-Cite 200DC (Excelitas) light source, and a DAPI filter cube.

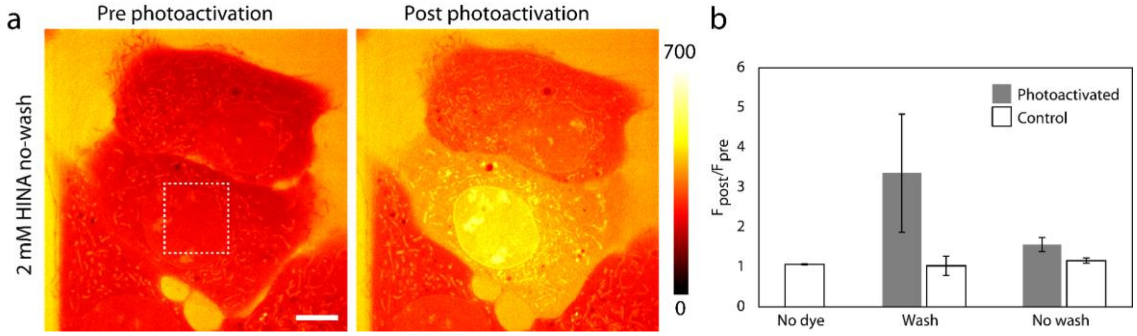

Figure S70. (a) Confocal images before and after photoactivation of U2OS cells incubated with HINA (2 mM) without washing. Dashed boxes indicate the area that was irradiated with UV. Color bars and corresponding minimum and maximum number of counts in displayed in the image are on the right. Scale bars: 10 µm. (b) Quantification of fluorescence intenstity in the different conditions in the cells irradiated with UV light (photoactivated, gray) and in neighbouring, non-irradiated cells (control, white). N=2 for no dye, N=3 for wash (glow up was observed in 3/5 cells. Values shown are the average of the responding cells only), N=5 for no wash (glow up observed in all tested cells). See also the main text Figure 3.

14.2. Photoactivation test in solvent

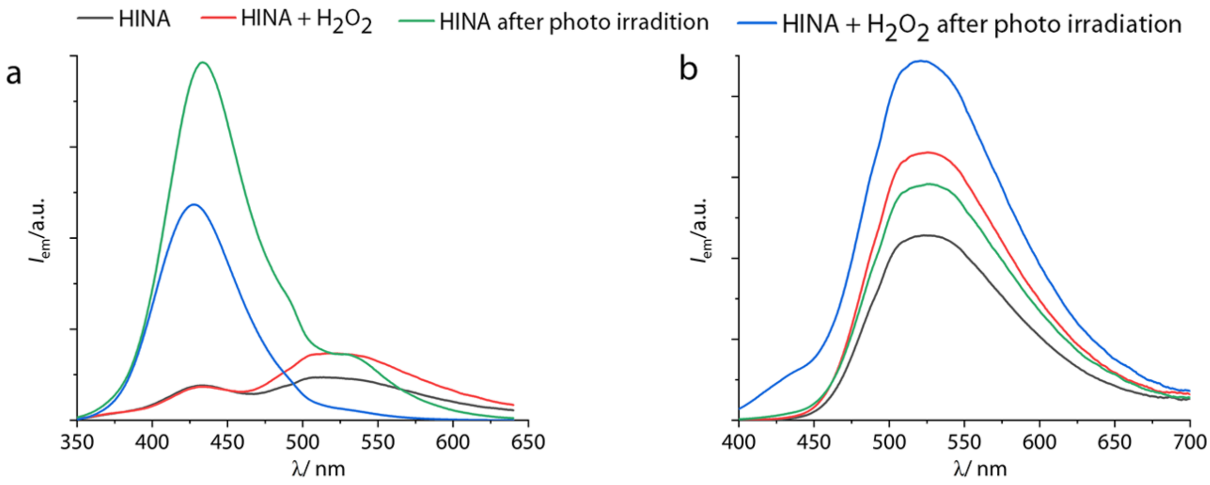

Figure S71. Emission spectra of HINA (1 mM, black line), HINA upon addition of H_2O_2 (10 mM, red line), the mixture of HINA and H_2O_2 with photo irradiation 30 min (blue line) and HINA alone with photo irradiation 30 min (green line). a) the exciation wavelength is 330 nm; in b) is 380 nm. All the spectra were measured in PBS buffer at pH 6.

15. Computational results

Coupled-cluster calculations were performed using the second-order approximate coupled-cluster singles-and-doubles model CC2[13] in the def2-TZVPPD basis set[14], using the resolution-of-the-identity (RI) approximation with corresponding auxiliary basis set (cbas)[15, 16]. The 1s core orbitals were not included in the CC2 calculations (frozen-core approximation). All calculations were performed with version V7.4.0 of the TURBOMOLE program package.[17] Geometry optimizations of ground and excited states were performed with tight thresholds (scfconv=10, denconv=1d-9, jobex -level cc2 -energy 9 -gcart 6) and harmonic vibrational frequencies were obtained from numerical differences of analytical nuclear gradients using the NumForce script (NumForce -level cc2 -central -d 0.001). Franck-Condon profiles were computed using the time-dependent wave-packet approach as implemented in hotFCHT.[18] These computations were performed for a temperature of 298.15 K and include a Gaussian broadening with full width at half maximum of 500 cm^{-1}. Large discrepancies with the experiments were found when computing the emission transitions for the neutral forms of HINA and its analogues due to very different equilibrium structures of ground and excited states, which can be interpreted as an excited-state intramolecular proton-transfer process (ESIPT). Figure S73 shows the ground and excited state energies of HINA along a scan ("reaction coordinate"), which is obtained from a linear interpolation between the ground-state equilibrium structure (100%) and the excited-state equilibrium structure (0%).

Table S20. Vertical absorption (VA) energy (in eV) with corresponding oscillator strength (f_{osc}) vertical emission (VE) energy (in eV) with corresponding oscillator strength (f_{osc}) adiabatic electronic energy (in eV) and 0-0 transition (in eV) of the $n \to \pi^*$ and $\pi \to \pi^*$ transitions of the anions of HINA, SA, and HPA. Obtained at the CC2/def2-TZVPPD level. Wavelengths (in nm) are given in parentheses.

		VA	f_{osc}	VE	f_{osc}	Adiab.	0-0
**HINA**	$n \to \pi^*$	2.81 (442)	0.0003	1.78 (696)	0.0001	2.38 (521)	2.31 (537)
	$\pi \to \pi^*$	2.99 (415)	0.1669	2.20 (562)	0.0889	2.66 (466)	2.59 (479)
**SA**	$n \to \pi^*$	3.09 (402)	0.0001	1.99 (624)	0.0001	2.60 (476)	2.51 (495)
	$\pi \to \pi^*$	3.09 (402)	0.1641	2.47 (502)	0.0985	2.28 (440)	2.73 (453)
**HPA**	$n \to \pi^*$	2.74 (453)	0.0002	1.84 (674)	0.0001	2.35 (527)	2.27 (547)
	$\pi \to \pi^*$	3.03 (409)	0.1582	2.36 (525)	0.0844	2.74 (452)	2.67 (464)

Table S21. Leading natural transition orbitals (NTOs) of the $n \rightarrow \pi^*$ and $\pi \rightarrow \pi^*$ transitions of the anions of HINA, SA, and HPA, computed at the equilibrium geometries of the respective excited states. Obtained at the CC2/def2-TZVPPD level and plotted for an isosurface value of $\pm 0.05\ a_0^{-3/2}$.

	$\boldsymbol{n \rightarrow \pi^*}$		$\boldsymbol{\pi \rightarrow \pi^*}$	
	**Hole state**	**Particle state**	**Hole state**	**Particle state**
**HINA**	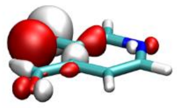	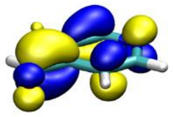	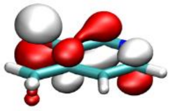	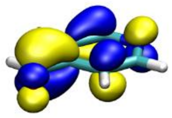
**SA**	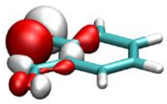	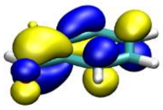	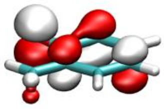	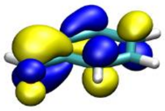
**HPA**	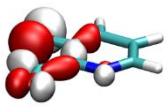	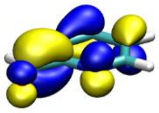	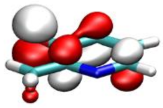	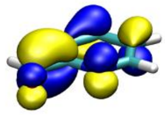

Table S22. Colors (RGB values) obtained from the computed emission spectra of Figure S67.

	$\boldsymbol{n \rightarrow \pi^*}$				$\boldsymbol{\pi \rightarrow \pi^*}$			
	**R**	**G**	**B**		**R**	**G**	**B**	
**HINA**	255	52	0		151	138	0	
**SA**	255	104	0		41	132	58	
**HPA**	255	40	0		84	141	26	

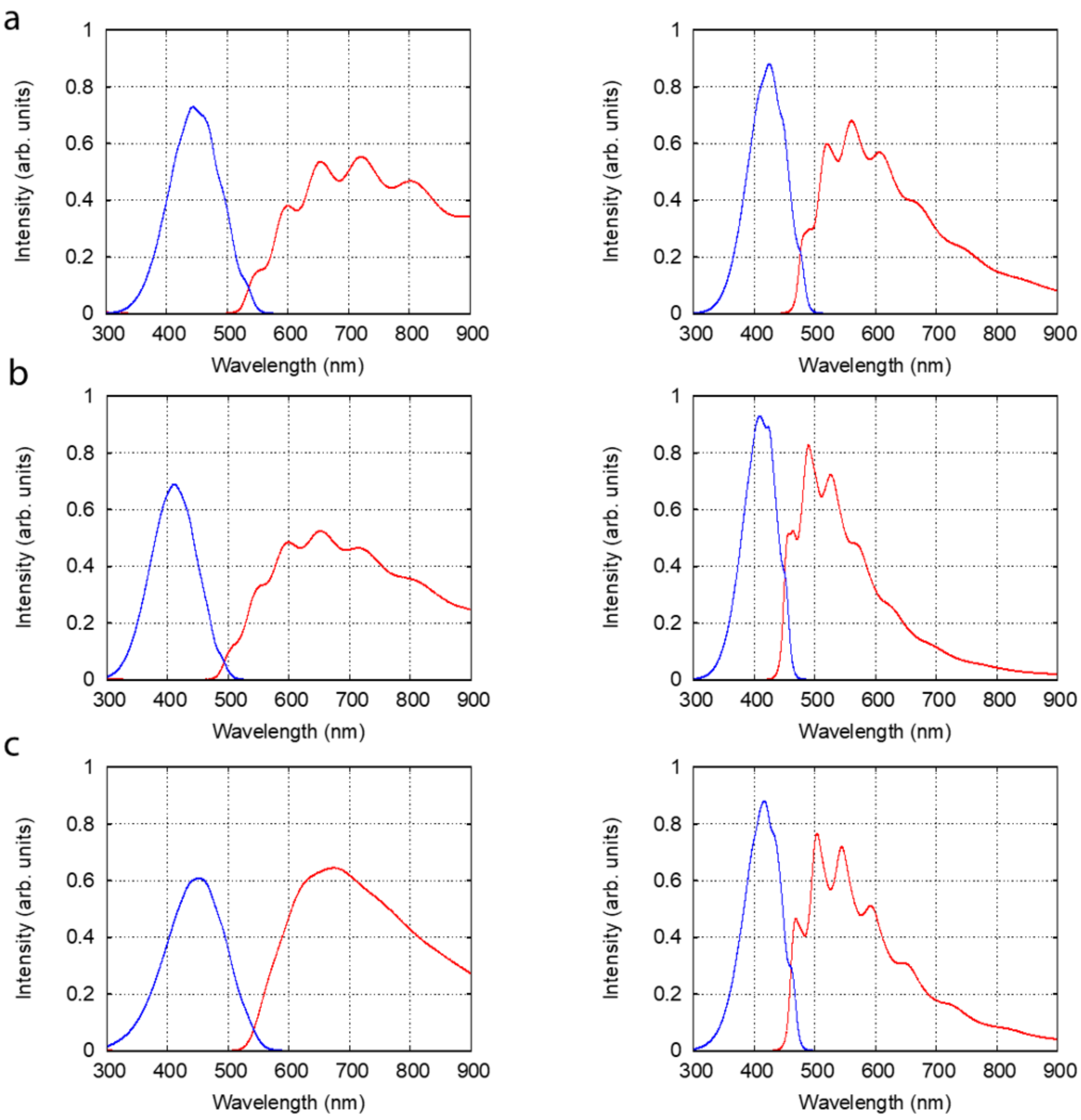

Figure S72. Franck-Condon profiles of absorption (blue lines) to and emission (red lines) from the $n \rightarrow \pi^*$ (left) and $\pi \rightarrow \pi^*$ (right) excited states of the anions of a) HINA, b) SA, and c) HPA. Computed at 298.15 K with Gaussian broadening with full width at half maximum of 500 cm^{-1} from CC2/def2-TZVPPD data using the time-dependent wave-packet approach as implemented in hotFCHT.

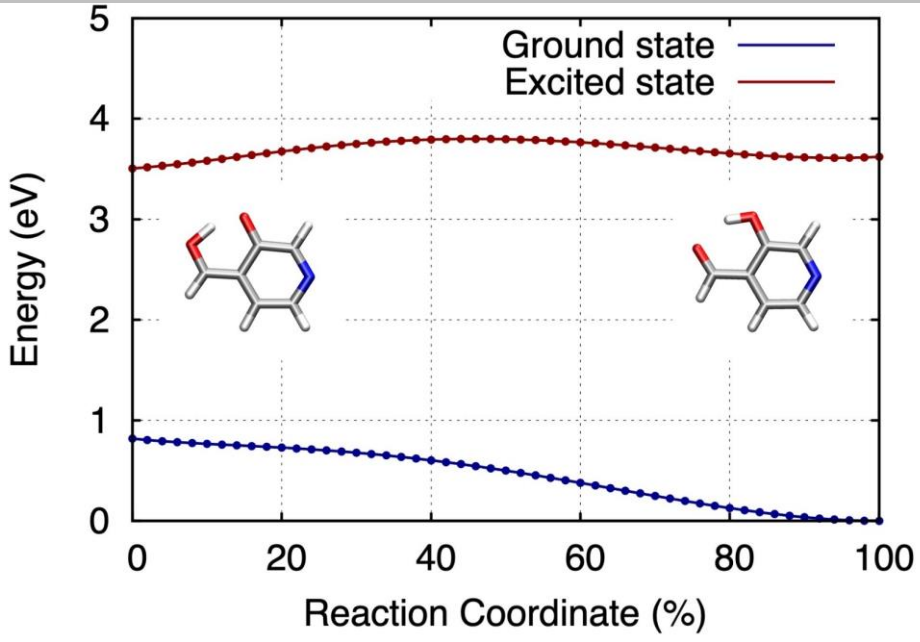

Figure S73. The potentical energy surface for the ground state (blue) and excited state (red) of HINA.

16. References

1. J. Zhou, H. Liu, B. Jin, X. Liu, H. Fu and D. Shangguan, *J. Mater. Chem. C*, 2013, **1**, 4427-4436.
2. J. Reijenga, A. van Hoof, A. van Loon and B. Teunissen, *Anal. Chem. Insights*, 2013, **8**, 53-71.
3. R. de Levie, *The Chemical Educator*, 1996, **1**, 1-18.
4. Using Excel to Fit a Titration Curve, http://www.uky.edu/~holler/che226/titrations/titration_curve.html#estimating, (accessed accessed 15 February 2020).
5. B. Szczepanik, *J. Mol. Struct.*, 2015, **1099**, 209-214.
6. L. M. Tolbert and K. M. Solntsev, *Acc. Chem. Res.*, 2002, **35**, 19-27.
7. A. S. M. Islam, M. Sasmal, D. Maiti, A. Dutta, B. Show and M. Ali, *ACS omega*, 2018, **3**, 10306-10316.
8. J. Krasowska, M. Olasek, A. Bzowska, P. L. Clark and B. Wielgus-Kutrowska, *J. Spectrosc. (Hindawi)*, 2010, **24**, 343-348.
9. M. Cortijo, J. Llor and J. M. Sanchezruiz, *J. Biol. Chem.*, 1988, **263**, 17960-17969.
10. S. H. Hilal, L. L. Bornander and L. A. Carreira, *QSAR Com. Sci.*, 2005, **24**, 631-638.
11. E. G. S. a. W. P. Jencks, *J. Am. Chem. Soc.*, 1968, 6154-6162.
12. K. Abe, H. Endo and M. Hirota, *Bull. Chem. Soc. Jpn.*, 1981, 466-469.
13. O. Christiansen, H. Koch and P. Jørgensen, *Chem. Phys. Lett.*, 1995, **243**, 409-418.
14. D. Rappoport and F. Furche, *J. Chem. Phys.*, 2010, **133**, 134105.
15. C. Hättig and F. Weigend, *J. Chem. Phys.*, 2000, **113**.
16. A. Hellweg and D. Rappoport, *Phys. Chem. Chem. Phys.*, 2015, **17**, 1010-1017.
17. Program Package For Electronic Structure Calculations, http://www.turbomole.com).
18. H. C. Jankowiak, J. L. Stuber and R. Berger, *J. Chem. Phys.*, 2007, **127**, 234101.
